# Stable and Unstable Concentration Oscillations Induced by Temperature Oscillations on Reversible Nonequilibrium Chemical Reactions of Helicene Oligomers

**DOI:** 10.3390/ijms24010693

**Published:** 2022-12-30

**Authors:** Sheng Zhang, Ming Bao, Mieko Arisawa, Masahiko Yamaguchi

**Affiliations:** 1 State Key Laboratory of Fine Chemicals, Dalian University of Technology, Dalian 116024, China; 2 Department of Bioscience and Biotechnology, Graduate School of Bioresource and Bioenvironmental Sciences, Kyushu University, Fukuoka 819-0395, Japan

**Keywords:** unstable concentration oscillation, temperature oscillation, reversible chemical reaction, helicene oligomer, stable concentration oscillation, nonequilibrium

## Abstract

Temperature oscillations can affect behaviors of living things. In this article, we describe the effect of triangle temperature oscillations on reversible nonequilibrium chemical reactions detected as concentration oscillations. When amplification through self-catalytic reactions is involved in the chemical reactions, concentration oscillations exhibit diverse nonequilibrium phenomena, which include equilibrium intersecting, equilibrium noncontact, and equilibrium sliding. Both stable and unstable concentration oscillations occur, during which repeated cycles provide the same and different concentration oscillations, respectively. Concentration oscillations are classified according to their waveforms in concentration/time profiles, the shapes of hysteresis curves in concentration/temperature profiles, the nature of self-catalytic reactions, and their relationships with equilibrium. An unstable concentration oscillation may be transformed into a stable concentration oscillation, which is described on the basis of the classifications. Experimental examples are shown using reversible association and dissociation reactions of helicene oligomers.

## 1. Introduction

### 1.1. Temperature Oscillations

Oscillation is the phenomenon of repetitive or periodic variation, typically in time, of some measure of a central value or between two or more different states [1,2,3]. Mechanical oscillations of a pendulum and alternating electric current are examples of this in physics. In our daily and yearly life, various environmental oscillations occur. Temperature oscillations occur with repeated cycles of high and low temperatures over days and years, which markedly affect the behavior of animals, plants, and microorganisms. For example, sakura flowers blossom in spring but not in autumn, which occurs at a certain temperature during a temperature increase but not a decrease [4,5]. Circadian rhythms coordinate behavior and physiology of organisms with environmental changes in day–night 24-h cycles, in which temperature oscillation can induce significant effects [6,7,8,9]. The mechanisms underlying flower blossoming and circadian rhythms have been studied at the levels of the molecule, cell, organ, and individual organism.

Among various environmental oscillations, of note are temperature oscillations. When living things respond to temperature oscillations at the molecular level, it is considered that the oscillations are converted into concentration oscillations via chemical reactions of biomacromolecules, such as nucleic acids and proteins, and the concentration oscillations are processed by complex biochemical systems for relevant biological responses. Such chemical reactions are considered to involve amplification mechanisms for high sensitivity, which are referred to as positive feedback. Thus, biological responses against environmental temperature oscillations are induced by complex biochemical systems, which contain chemical reactions highly sensitive to temperature oscillations. It is, therefore, critical to understand the effect of temperature oscillations on chemical reactions involving amplification, detected as concentration oscillation. 

In addition to implications in biology, studies on concentration oscillations induced by temperature oscillations may provide diverse applications in daily and yearly events. For example, daily concentration oscillations may provide a drug delivery system, with which the drug exerts effect at night or in the morning; yearly concentration oscillations may be employed for crops, the growth of which efficiently responds in spring. Application in information processing is also conceivable such as induction of an event after three days or three cycles of temperature oscillation. Temperature oscillations may also be used to control synthetic chemical reactions by the acceleration or enhancement of chemical yields. 

**Figure 1:**
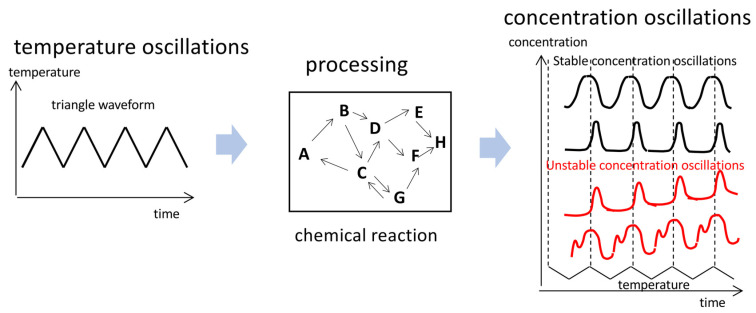
Input/output systems involving temperature oscillation inputs and stable/unstable concentration oscillation outputs via processing with chemical reaction.

The relationships of temperature oscillations and concentration oscillations can be described by the concept of input/output systems, in which temperature oscillation inputs are transformed into concentration oscillation outputs via processing with chemical reactions (Figure 1) [10,11].

Of note is that these concentration oscillation phenomena occur in response to temperature oscillations and are different from autonomous chemical oscillations, such as the Belousov–Zhabotinsky reaction, in which concentration oscillations occur at fixed temperatures [12,13,14,15].

### 1.2. Temperature and Concentration Oscillations

#### 1.2.1. Relationships between Temperature and Concentration Oscillations

Temperature oscillation is characterized by its amplitude, frequency, period, and waveform in a cycle, which is a single unit of oscillation [16]. Amplitude *is* the distance between high and low temperatures; frequency is the number of temperature oscillations per unit of time; period is the amount of time required for one complete cycle of temperature change; waveform is the shape of a graph showing the temperature change as a function of time. 

Waveforms in a temperature oscillation cycle include triangle, square, and sine [17]. The triangle waveform is so named for its triangular shape and is characterized by a periodic, piecewise linear, continuous real function. The square waveform has a constant-frequency amplitude alternating between fixed minimum and maximum values with the same duration at the minima and maxima. The sine waveform is any mathematical curve that describes a smooth periodic oscillation. 

In this article, we focus on concentration oscillations of chemical reactions caused by triangle temperature oscillations induced by cooling and heating at a constant rate between high and low temperatures with linear temperature changes versus time. Experimentally, temperature oscillations of a chemical reaction can conveniently be induced by the Peltier apparatus. The relationships of triangle temperature oscillations and concentration oscillations may be easier to consider than those of sine temperature oscillations because of the linear nature of temperature changes. Triangle temperature oscillations are simply noted “temperature oscillations” hereafter. Square temperature oscillations are applied in some cases. 

Concentration oscillations can be described by their amplitude, frequency, period, and waveform, and their relationships with temperature oscillations are defined by their phase and periodicity. Phase is used to compare temperature and concentration oscillations that occur simultaneously [18]. Same-phase oscillations indicate that maximum and minimum of temperature and concentration oscillations occur at the same temperature; phase shift implies a change in the maximum and minimum in concentration oscillations versus temperature oscillations. When every cycle of a concentration oscillation provides the same amplitude, frequency, period, and waveform, such a periodic phenomenon [19] is called a stable concentration oscillation in this article [20]. When a concentration oscillation shows different amplitudes, frequencies, periods, or waveforms in repeated cycles, such a pseudo-periodic phenomenon [21,22] is called an unstable concentration oscillation [20].

#### 1.2.2. Effect of Temperature Oscillations on Equilibrium

The effect of temperature oscillations on reversible and irreversible chemical reactions are briefly summarized in the following part of the introduction in Section 1. The latter include equilibrium and nonequilibrium reversible chemical reactions.

Reversible and irreversible chemical reactions occur depending on the relative thermodynamic stability of substrates and products. When the difference in the Gibbs free energy Δ*G* between substrates and products is negative with a large absolute value, the reaction irreversibly proceeds from substrates to products [23]. Temperature oscillations under ambient conditions induce changes in reaction rate but not the direction, and concentration does not oscillate.

Reversible chemical reactions are essential to concentration oscillations, in which substrates and products are switched under suitable conditions. In contrast to an irreversible chemical reaction, a reversible chemical reaction involves a relatively small difference in Δ*G*, and, accordingly, both direction and reaction rate can change owing to temperature oscillations. Reversible chemical reactions can be either at equilibrium or nonequilibrium, which results in different relationships between temperature and concentration oscillations.

**Figure 2:**
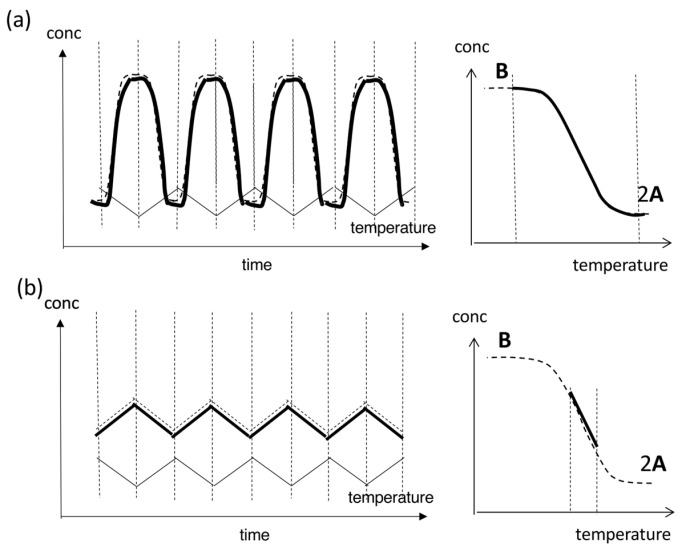
Stable concentration oscillations provided by temperature oscillations at equilibrium shown by concentration/time and concentration/temperature profiles in (**a**) sinusoidal concentration oscillations (SE-1) in the sigmoidal domain of equilibrium curve containing two limit states and (**b**) triangle concentration oscillations (SE-2) in the linear domain. Solid thin lines indicate temperature oscillation; dashed lines indicate equilibria.

Behaviors of an equilibrium reaction, which is a typical reversible chemical reaction under temperature oscillations, can be described by thermodynamics. Consider an equilibrium 2**A**⇆**B**, in which **B** is thermodynamically stable at low temperatures and 2**A** at high temperatures [23]. Forward and backward reaction rates are equal, and the concentrations of **A** and **B**, [**A**] and [**B**], are fixed at a fixed temperature. The equilibrium constant *K* is described by *K* = [**B**]/[**A**]^2^ with the total concentration [**A**]_0_ = [**A**] + 2[**B**]. *K* is related to the absolute temperature *T* by *K* = exp(−Δ*G*/*RT*), in which *R* is the gas constant and Δ*G* is the difference in the Gibbs free energy between 2**A** and **B**. Increases and decreases in the *T* exert monotonic sigmoidal changes in [**B**] according to *K* = exp(−Δ*G*/*RT*), which is shown by the concentration/temperature profiles (Figure 2a). An equilibrium occurs involving the same pathway of 2**A**→**B** and **B**→2**A** during cooling and heating.

When temperature oscillation is provided to the equilibrium 2**A**⇆**B**, concentration instantaneously changes to achieve other equilibria immediately. Then, concentration oscillations with a sinusoidal waveform along the sigmoidal equilibrium curve occur, as shown in the concentration/time profile (Figure 2a). A stable concentration oscillation occurs in cycles with the same amplitude, frequency, period, and waveform. The concentration changes proceed through the same pathway during cooling and heating, and the waveform accordingly is symmetric. The phase in concentration oscillations is identical to that in temperature oscillations. This oscillation phenomenon is named SE-1 in this article.

SE-1 appears when the temperature range in temperature oscillations is broad, containing high- and low-temperature limit states, which are defined here as flat domains in sigmoidal curves at the high- and low-temperature extremes, respectively (Figure 2a). When the range in temperature oscillations is narrow at the intermediate linear state of the sigmoidal curve, a concentration oscillation with a triangle waveform appears, as shown in the concentration/time profile (Figure 2b), and the oscillation is named SE-2.

### 1.3. Effect of Temperature Oscillations on Reversible Nonequilibrium Chemical Reactions

The effect of temperature on nonequilibrium chemical reactions has generally been studied by observing the kinetics of the reactions under fixed-temperature (isothermal) conditions [23]. In contrast, the effect of temperature oscillations on nonequilibrium chemical reactions is not well understood. Kinetics of the reactions under temperature change (nonisothermal) conditions are analyzed by empirical methods, as shown in thermogravimetric analysis (TGA) and differential scanning calorimetry (DSC) studies [24,25,26]. When a temperature oscillation is applied to a chemical reaction, complex nonisothermal kinetics can appear. Note that chemical kinetics under isothermal conditions may not directly be applicable to nonisothermal conditions [27,28].

**Figure 3:**
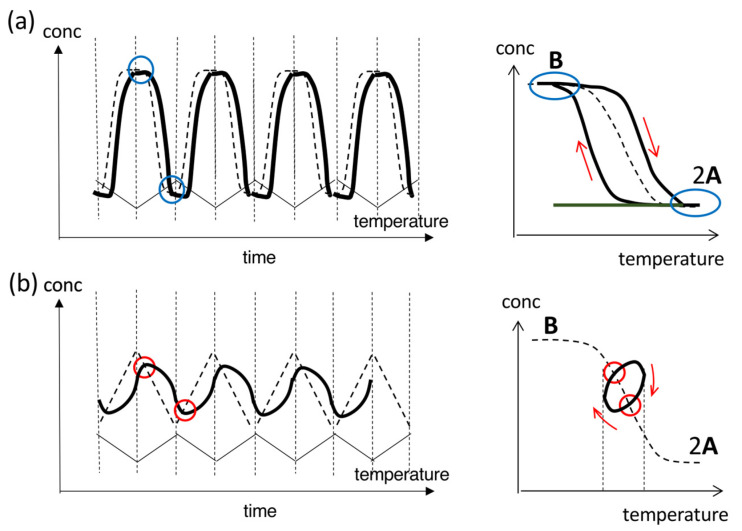
Stable concentration oscillations with delay hysteresis shown by bold black lines: (**a**) Sinusoidal concentration oscillations with normal hysteresis involving overlapping with the equilibrium curve (equilibrium overlapping) (SD-1) and (**b**) sinusoidal concentration oscillation with oval hysteresis involving intersecting with the equilibrium curve (equilibrium intersecting) (SD-2). Solid thin lines indicate temperature oscillation; blue circles indicate equilibrium overlapping; red arrows indicate directions of hysteresis; red circles indicate equilibrium intersecting; dashed black lines indicate equilibria.

Reversible nonequilibrium chemical reactions can occur involving different pathways. Consider a reversible chemical reaction between 2**A** and **B**, in which 2**A**→**B** and **B**→2**A** occur under cooling and heating, respectively. When concentration oscillation is delayed with respect to temperature oscillation, different phenomena occur during 2**A**→**B** and **B**→2**A** beyond equilibrium. Such a reversible nonequilibrium chemical reaction is noted here as 2**A**
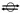
**B** to discriminate them from those at equilibrium 2**A**⇆**B** [29]. In such a case, concentration oscillations involving 2**A**
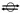
**B** result in different cooling and heating curves in the concentration/temperature profiles (Figure 3), which are in contrast to those involving 2**A**⇆**B**, resulting in the same cooling and heating curves (Figure 2). Accordingly, the waveform is unsymmetric, as shown in the concentration/time profiles, and a phase shift occurs, in which maxima and minima in temperature and concentration oscillations in time do not coincide. This phenomenon in concentration/time profiles is called hysteresis and occurs in nonequilibrium systems [30]. In the following discussions, reversible nonequilibrium chemical reactions are simply called “reactions”.

Both concentration/time and concentration/temperature profiles are used to describe the relationships between concentration and temperature oscillations. Concentration/time profiles exhibit time courses of concentration oscillations showing amplitude, frequency, period, waveform, and phase shift. Concentration/temperature profiles are used to demonstrate hysteresis by comparison of concentration changes during cooling and heating and also cooling and heating curves with the equilibrium curve: these comparisons reveal qualitative distances from equilibrium. Note that concentration/time and concentration/temperature profiles are in one-to-one correspondence when a single reaction 2**A**
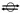
**B** with two species **A** and **B** involved.

This article attempts to discuss the effect of temperature oscillations on reversible nonequilibrium chemical reactions. Emphasized are chemical reactions involving self-catalytic reactions, which significantly amplify subtle changes in concentration. Concentration oscillations against temperature oscillations are classified and are employed to describe experimental stable and unstable concentration oscillations. The experimental results obtained in our previous studies are explained based on the classification. Such analysis of chemical reactions has not systematically been discussed before.

The following subjects are addressed in this article: Section 1 provides an introduction to concentration oscillations caused by temperature oscillation involving chemical reactions. Section 2 and Section 3 provide general discussions on stable and unstable concentration oscillations, respectively. Section 4, Section 5 and Section 6 provide experimental examples of concentration oscillations involving delay and amplified hysteresis. Section 7 provides an extension of unstable concentration oscillations to the study of the nature of a reaction. Section 8 provides conclusions.

## 2. Stable Concentration Oscillations

Stable concentration oscillations occur with the same amplitude, frequency, period, and waveform in repeated cycles, which result in the same state at the same temperature with regard to concentration, reaction rate, and direction of reactions. Stable concentration oscillations are in contrast to unstable concentration oscillations with different amplitudes, frequencies, periods, or waveforms in repeated cycles, which do not result in the same state at the same temperature. General discussions on stable concentration oscillations are provided in Section 2, and those of unstable concentration oscillations will be provided in Section 3.

### 2.1. Delay and Amplify Hysteresis

Stable concentration oscillations SE-1 and SE-2 occur by equilibrium 2**A**⇆**B**, as described in Section 1.3, which involves the same reaction pathways during cooling and heating, because equilibrium is thermodynamically the most stable state at a given temperature (Figure 2). Stable concentration oscillations can also occur in reversible nonequilibrium reaction 2**A**
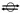
**B**, involving a delay against temperature oscillations, which show hysteresis owing to different delays during cooling and heating [29]. Such hysteresis due solely to a delay is named here delay hysteresis (Figure 3).

**Figure 4:**
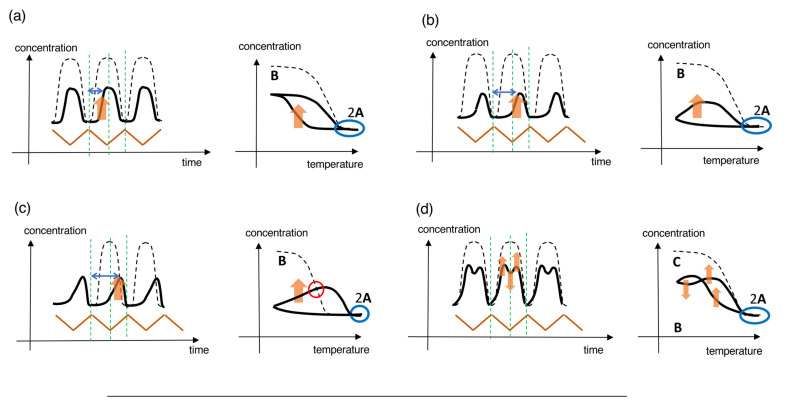

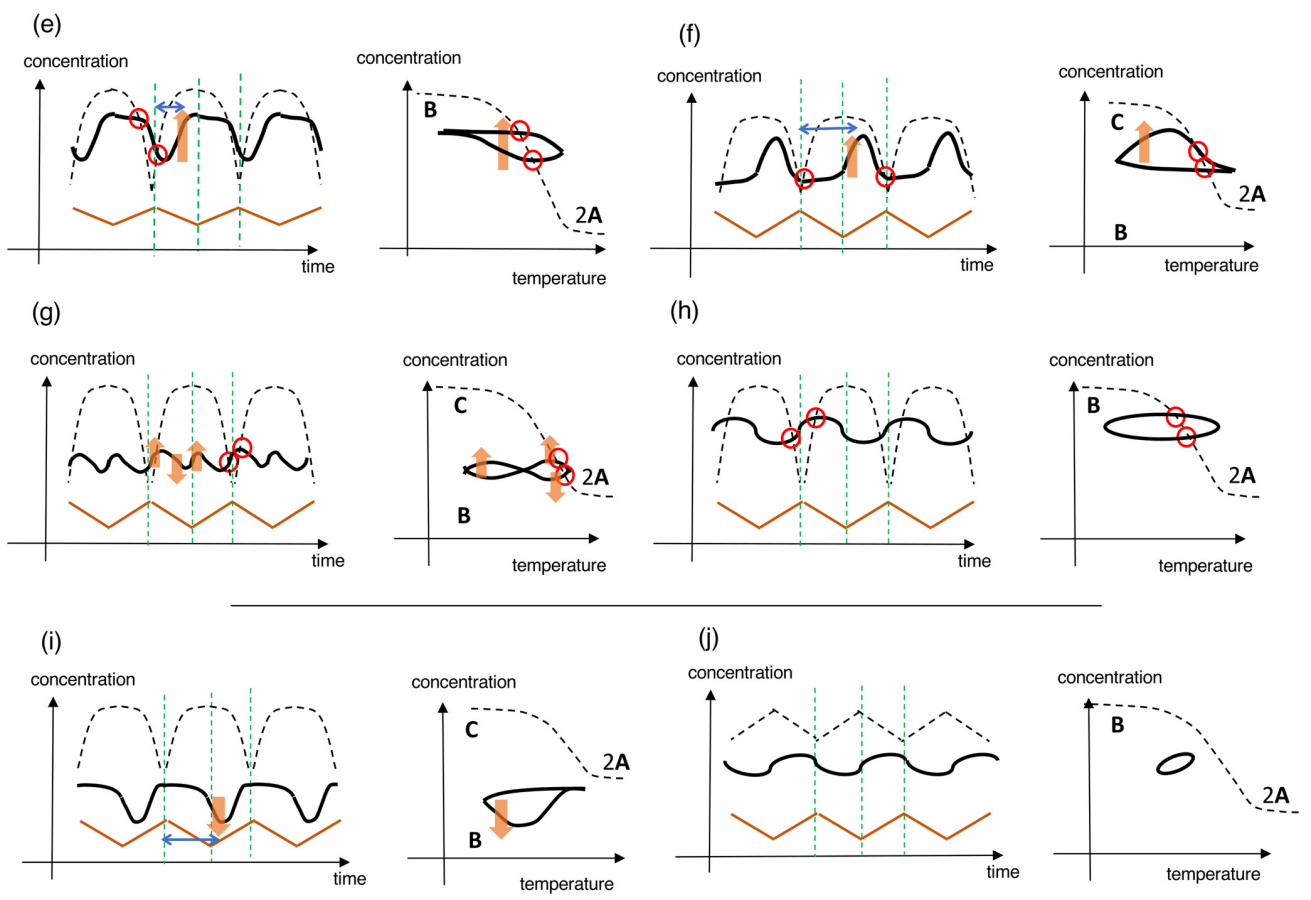
Classification of stable concentration oscillations involving 2**A** + **B**→2**B** and/or 2**A** + **C**→2**C**. Stable concentration oscillations with amplify hysteresis involving equilibrium overlapping: (**a**) Sinusoidal concentration oscillations with semi-normal hysteresis (SAO-1), (**b**) retarded concentration oscillations with inflation hysteresis (SAO-2), (**c**) retarded concentration oscillations with inflation hysteresis (equilibrium crossing) (SAO-3), and (**d**) frequency-doubled concentration oscillations with figure-eight hysteresis (SAO-4). Stable concentration oscillations with amplify hysteresis involving intersecting with the equilibrium curve (equilibrium intersecting): I sinusoidal concentration oscillations with semi-normal hysteresis (SAI-1); (**f**) retarded concentration oscillations with inflation hysteresis (SAI-2); (**g**) frequency-doubled concentration oscillations with figure-eight hysteresis (SAI-3); (**h**) sinusoidal concentration oscillations with long oval hysteresis intersecting the equilibrium curve (equilibrium touching) (SAI-4). Stable concentration oscillations with amplify hysteresis involving not contacting the equilibrium curve (equilibrium noncontact): (**i**) Retarded concentration oscillations with inflation hysteresis (SAN-1) and (**j**) sinusoidal concentration oscillations with oval hysteresis (SAN-2). Blue circles indicate equilibrium overlapping; orange bold arrows indicate self-catalytic reactions; red circles indicate intersection of concentration oscillation curves and equilibrium curves; dashed lines indicate equilibrium curves; blue arrows in (**a**–**c**,**e**,**f**,**i**) indicate phase shifts; green vertical dashed lines show switching between cooling and heating and are provided to show the unsymmetric nature of waveforms.

Another type of hysteresis that occurs owing to delay and also to amplification in 2**A**
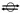
**B** is named amplify hysteresis (Figure 4). We previously developed helicene oligomers, which exhibit amplify hysteresis during the interconversion between double-helix **B** and random-coils 2**A** [29,31,32,33,34]. In particular, amplification is induced by self-catalytic reaction 2**A** + **B**→2**B**, which exhibits extremely high sensitivity to temperature changes. Increases in the concentration of the product **B**, which is also a catalyst, with the progress of the reaction, significantly accelerate 2**A** + **B**→2**B** [32]. Section 2.2 and Section 2.3 provide general discussions on stable concentration oscillations with delay and amplify hysteresis, respectively.

### 2.2. Stable Concentration Oscillations with Delay Hysteresis

Stable concentration oscillations with delay hysteresis appear when the oscillations occur with delay against temperature oscillations (Figure 3). Delay hysteresis involving 2**A**
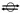
**B** is described by comparing temperature change rate and reaction rate. Consider a reaction 2**A**→**B** initiated by cooling of 2**A** at high temperatures under equilibrium. When 2**A**→**B** is very slow versus cooling, no reaction occurs, which results in a horizontal straight line without changes in concentration, as shown in the concentration/temperature profile (Figure 3a right, green line). When 2**A**→**B** is very fast versus cooling or occurs instantaneously, the cooling curve coincides with the equilibrium curve (Figure 2a). When 2**A**→**B** is modestly slow versus cooling, the initial delay is followed by 2**A**→**B** to reach equilibrium at low temperatures, which results in a sigmoidal cooling curve with high- and low-temperature limit states (Figure 3a, black line). Note that 2**A**→**B** must override retardation during cooling. Delay also occurs during heating in **B**→2**A**. Then, cooling and heating result in different curves on both sides of the equilibrium curve where the system is out of equilibrium (Figure 3a); this behavior is called normal hysteresis, previously referred to as both-side hysteresis [35]. A small phase shift appears in a sinusoidal concentration oscillation versus a temperature oscillation, as shown in the concentration/time profile. The phenomenon involving sinusoidal concentration oscillation with normal hysteresis is named SD-1.

When cooling and heating curves overlap with the equilibrium curve at high- and/or low-temperature limit states, the phenomenon is named equilibrium overlapping (Figure 3a, blue circles). Equilibrium overlapping of SD-1 allows the system to reach an equilibrium state, and the subsequent cycle provides the same cooling and heating curves. Then, a stable concentration oscillation occurs. Theoretical analysis of SD-1 with delay hysteresis has been conducted, taking advantage of its relatively simple nature [36,37].

SD-1 occurs under temperature oscillations over a broad temperature range including high- and low-temperature limit states (Figure 3a). When temperature oscillations over a narrow range at the linear part of the equilibrium curve are applied, a small amplitude of sinusoidal concentration oscillation with oval hysteresis appears, which is named SD-2 (Figure 3b). Oval hysteresis is derived from the competition between 2**A**→**B** and **B**→2**A** with delay. Upon heating, [**B**] increases after intersecting the equilibrium curve caused by delay, which is against the lower thermodynamic stability of **B** than of 2**A** at high temperatures. Then, the maximum [**B**] is reached owing to overshooting, and then [**B**] starts to decrease. Upon cooling, the decrease in [**B**] is followed by an increase with delay because of the higher thermodynamic stability of **B** than of 2**A** at low temperatures. The minimum [**B**] is reached owing to undershooting, and then [**B**] starts to increase. The cooling and heating curves intersect with the equilibrium curve twice during cooling and heating at the central part. The intersecting phenomenon of [**B**] curves with the equilibrium curve is named equilibrium intersecting. One difference between SD-1 and SD-2 is temperature oscillation ranges; SD-1 occurs at a sinusoidal region of the equilibrium curve and SD-2 at a linear region. Another difference is the occurrence of equilibrium overlapping in SD-1 and equilibrium intersecting in SD-2, the details of which will be discussed in Section 2.3.3.

To summarize Section 2.2, temperature oscillations provide stable concentration oscillations with delay hysteresis, which involves the same amplitude, frequency, period, and waveform in repeated cycles. SD-1 occurs with temperature oscillations over a broad temperature range and exhibits sinusoidal concentration oscillations with normal hysteresis; SD-2 occurs with temperature oscillations over a narrow temperature range and involves sinusoidal concentration oscillations with oval hysteresis (Figure 3). Delay hysteresis of SD-1 and SD-2 occurs close to equilibrium because of the lack of amplification, which is in contrast to amplify hysteresis, as will be discussed in Section 2.3.

### 2.3. Stable Concentration Oscillations with Amplify Hysteresis

Stable concentration oscillations with amplify hysteresis caused by 2**A**
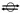
**B** involves delay against temperature oscillations and also amplification in reactions (Figure 4). In particular, amplification through the self-catalytic reaction 2**A** + **B**→2**B** is considered here, in which the product **B** catalyzes the reaction of 2**A** to form **B**, and the concentration of **B** significantly increases with the progress of the reaction. The self-catalytic reaction 2**A** + **B**→2**B** is highly sensitive to conditions such as the temperature range, high/low temperatures, rate of temperature change, concentration, and thermal history. In addition, 2**A**
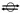
**B** involves markedly different mechanisms of 2**A**→**B** and **B**→2**A**. In order to understand such phenomena of stable concentration oscillations, they are classified according to their waveforms in concentration/time profiles, the shapes of hysteresis curves in concentration/temperature profiles, the nature of the self-catalytic reaction, and their relationships with equilibrium. The relationships of stable concentration oscillations with equilibrium are expressed by their overlapping, intersecting, and noncontact with the equilibrium curve. The complex nature of stable concentration oscillations with amplify hysteresis is contrasted to the relatively simple nature of the oscillations with delay hysteresis (Figure 3).

#### 2.3.1. Stable Concentration Oscillations Involving Equilibrium Overlapping

Stable concentration oscillations with amplify hysteresis caused by 2**A**
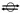
**B** involving 2**A** + **B**→2**B** is described herein, in which **A** is thermodynamically stable at low temperatures and **B** at high temperatures. Upon cooling of 2**A** in the high-temperature equilibrium state, 2**A** + **B**→2**B** occurs with delay accompanied by a significant increase in [**B**], whose formation overrides retardation upon cooling owing to amplification through 2**A** + **B**→2**B** (Figure 4a right). Further cooling retards the formation of **B**, and a steady state is reached at low temperatures. Heating induces **B**→2**A**, in which an equilibrium state containing 2**A** is reached at high temperatures. Sigmoidal cooling and heating curves then appear, named semi-normal hysteresis. Sinusoidal concentration oscillation is accompanied by a phase shift, as shown in the concentration/time profile (Figure 4a left), which is named SAO-1. SAO-1 occurs on one side of the equilibrium curve, which differs from SD-1 occurring on both sides (Figure 3a). In addition, of note is that equilibrium overlapping occurs in SAO-1 at high-temperature limit states (Figure 4a right, blue circle), which provides a stable concentration oscillation because equilibrium is achieved.

When [**B**] increases during heating with significant delay, retarded concentration oscillation and inflation hysteresis appear, which is named SAO-2 (Figure 4b). In contrast to SAO-1 involving an increase in [**B**] during cooling (Figure 4a, orange bold arrows), SAO-2 shows such an increase during heating (Figure 4b, orange bold arrows), which results in a large phase shift in the retarded concentration oscillation. The phase shifts in SAO-1 and SAO-2 (Figure 4a,b, blue arrows) are compared in terms of the increase in [**B**] with 2**A** + **B**→2**B** on the left and right sides of the central dashed vertical green lines, respectively, which indicate the switching from cooling and heating (Figure 4a,b, orange bold arrows). Inflation hysteresis results in a flat cooling curve and an inflation heating curve (Figure 4b right). Equilibrium overlapping at high temperatures results in a stable concentration oscillation (Figure 4b right, blue circle).

We previously described phenomenon called equilibrium crossing, in which the heating curve intersects with the equilibrium curve and significantly moves away to the other side of equilibrium (Figure 4c) [38]. This is due to 2**A** + **B**→2**B**, which occurs close to equilibrium and proceeds in the direction away from equilibrium (Figure 4c, orange bold arrows). When equilibrium overlapping occurs at high temperatures, a stable concentration oscillation occurs (Figure 4c, blue circle). The phenomenon is named SAO-3, which involves a retarded concentration oscillation with inflation hysteresis accompanied by equilibrium intersecting. Note that both equilibrium intersecting and equilibrium overlapping occur in SAO-3, which is in contrast to SAO-2, which lacks equilibrium intersecting (Figure 4b).

Frequency-doubled concentration oscillation with figure-eight hysteresis occurs as shown in the concentration/temperature profile, which exhibits two concentration maxima in a cycle in the concentration/time profile (Figure 4d). Figure-eight hysteresis involves the crossing of cooling and heating curves in a cycle. When equilibrium overlapping is involved, a stable concentration oscillation occurs (Figure 4d, blue circle). This behavior, named SAO-4, is derived from the competition between 2**A**
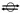
**B** and 2**A**
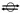
**C** involving 2**A** + **B**→2**B** and 2**A** + **C**→2**C**, respectively, (Figure 4d, orange arrows) in which **C** is thermodynamically stable at low temperatures.

With regard to the classification of hysteresis, gas absorption phenomena in heterogeneous systems have been reported [39,40]. In the present article, we provide another classification of hysteresis involving self-catalytic reactions in homogeneous solutions.

#### 2.3.2. Stable Concentration Oscillations Involving Equilibrium Intersecting

Other types of stable concentration oscillations, which do not involve equilibrium overlaying, are described in Section 2.3.2 and Section 2.3.4. Stable concentration oscillations can occur when the same states are provided at the same temperature in repeated cycles with regard to concentrations, direction of reactions, and reaction rates, and then the same subsequent cycle occurs. Such stable concentration oscillations occur when there is a balance between reactions and temperature change, and this situation is simply referred to “balanced state” in this article.

We classify stable concentration oscillations with balanced states in Section 2.3.2 when there is equilibrium intersecting but not equilibrium overlapping. Equilibrium intersecting is a phenomenon in which the cooling and heating curves intersect with the equilibrium curve. Different mechanisms of stable concentration oscillations involving equilibrium intersecting and equilibrium overlapping will be compared in Section 2.3.3.

Stable sinusoidal concentration oscillations with semi-normal hysteresis involving equilibrium intersecting are named SAI-1 (Figure 4e, red circles), and 2**A** + **B**→2**B** occurs during cooling (Figure 4e, orange arrows), which is analogous to SAO-1 (Figure 4a). However, one difference from SAO-1 is that SAI-1 involves equilibrium intersecting, whereas SAO-1 involves equilibrium overlapping.

A retarded concentration oscillation with inflation hysteresis, which is accompanied by equilibrium intersecting, is named SAI-2 (Figure 4f, red circles). Of note is that 2**A** + **B**→2**B** occurs during heating with significant delay (Figure 4f, blue and orange bold arrows), which contrasts to SAI-1 during cooling (Figure 4e, blue and orange arrows). Additionally, 2**A** + **B**→2**B** proceeds in the direction away from the equilibrium state containing **C**. In addition, of note is that both SAI-1 and SAO-2 involve 2**A** + **B**→2**B** with significant delay but are different because equilibrium overlapping and intersecting are involved, respectively.

The phenomenon of frequency-doubled concentration oscillation with figure-eight hysteresis accompanied by equilibrium intersecting is named SAI-3 (Figure 4g). Two concentration maxima appear in a cycle, as shown by the concentration/time profile, and cooling and heating curves cross in the concentration/temperature profile (Figure 4g, red circles). SAI-3 occurs owing to the involvement of competitive 2**A** + **B**→2**B** and 2**A** + **C**→2**C** (Figure 4g, orange arrows), and its mechanism will be discussed in Section 6.2.4. Despite the complex nature of the reaction system, balanced state is achieved, which demonstrates stable concentration oscillations. SAI-3 and SAO-4 show frequency-doubled concentration oscillations with different natures involving equilibrium intersecting and equilibrium overlapping, respectively.

When a self-catalytic reaction is minimal, a sinusoidal concentration oscillation with a long oval hysteresis appears with a small hysteresis area, which is named SAI-4 (Figure 4h). Equilibrium intersecting occurs during cooling and heating at high temperatures (Figure 4h, red circles). SAI-4 differs from SD-2 in terms of the narrow temperature ranges in temperature oscillations.

#### 2.3.3. Equilibrium Overlapping and Equilibrium Intersecting

Equilibrium overlapping and intersecting occur involving different modes of contact of cooling and heating curves with equilibrium curves: SAO-1 to SAO-4 involve equilibrium overlapping, in which their curves approach to the equilibrium curve in a tangential direction at high-temperature limit states and overlap with the equilibrium curve over a range of temperatures and a period of time (Figure 4a–d); SAI-1 to SAI-4 involve equilibrium intersecting, in which both their curves intersect with the equilibrium curve at points over a very small range of temperatures and a very short period of time (Figure 4e–h).

Equilibrium overlapping results in an equilibrium state over the overlapping range, in which the Boltzmann distribution and the detailed balance are achieved. The detailed balance is a concept of equilibrium, in which the forward and backward reaction rates are equal [41]. Equilibrium overlapping can provide a sufficient period of time to achieve equilibrium with the detailed balance. Equilibrium overlapping provides stable concentration oscillations, because the equilibrium loses memory of previous cycles, and the same subsequent cycles occur. This is because equilibrium is thermodynamically the most stable state at a given temperature and can be reached by diverse pathways.

In contrast, equilibrium intersecting does not reach equilibrium at the intersection, and overshooting occurs, in which the reaction proceeds beyond the equilibrium. The intersection points are not at equilibrium but in pseudo-equilibrium. Because the detailed balance is not achieved, the forward and backward reaction rates are not equal, although their concentrations are in agreement with the Boltzmann distribution. A sufficient period of time is not provided to achieve equilibrium with the detailed balance. However, of note is that stable concentration oscillations involving equilibrium intersecting can occur under the balanced state of reactions and temperature changes, examples of which will be described in Section 6.1.3.

#### 2.3.4. Stable Concentration Oscillations Involving Equilibrium Noncontact

Stable concentration oscillations can occur even without contact with the equilibrium curves, a situation named equilibrium noncontact. These oscillations occur out of equilibrium throughout a cycle when a balanced state is achieved and may involve self-catalytic reactions exhibiting amplify hysteresis.

Retarded concentration oscillation with inflation hysteresis involving equilibrium noncontact is named SAN-1, in which cooling and heating curves appear away from the equilibrium curve (Figure 4i). A large phase shift appears as 2**A** + **B**→2**B** occurs during heating with significant delay (Figure 4i, blue and orange bold arrows). Of note is that 2**A** + **B**→2**B** proceeds in the direction away from the equilibrium state containing **C**. Although SAN-1 occurs through a mechanism similar to those for SAO-2 and SAO-3 (Figure 4b,c), it involves equilibrium noncontact but not equilibrium overlapping nor intersecting.

Sinusoidal concentration oscillations with oval hysteresis can occur involving equilibrium noncontact, which is named SAN-2 (Figure 4j). SAN-2 occurs with minimal self-catalytic reactions and does not involve equilibrium intersecting as appears in SAI-4.

Temperature oscillations may provide various types of stable concentration oscillations with amplify hysteresis involving self-catalytic reactions. The relationships of cooling and heating curves with equilibrium curve are described by equilibrium overlapping, equilibrium intersecting, and equilibrium noncontact. Stable concentration oscillations can occur involving equilibrium overlapping upon achieving equilibrium and also can occur involving equilibrium intersecting and noncontact under the balanced states. The above classifications are examples of stable concentration oscillations, and many other types are conceivable, which are a subject in the future.

#### 2.3.5. Resonance Phenomenon

Resonance is a phenomenon involving the selective response of an object or a system that oscillates, induced by an externally applied oscillatory force [42,43]. When stable concentration oscillations with selective responses in terms of amplitude, frequency, period, or waveform occurs under a certain condition of temperature oscillation, such phenomenon is called resonance. A small change in temperature oscillation with regard to rate and range can exert a significant change in concentration oscillation through self-catalytic reactions. The resonance can also occur in unstable concentration oscillations. Experimental examples will be shown in Section 5 and Section 6.

Concentration oscillations can be considered as output in input/output systems, which include temperature oscillation input (Figure 1). Such systems can involve processing by reactions that convert thermal information to concentration information. Then, reactions can be used for sensing, signaling, and adaptation, which are common functions as in biological systems. For examples, SAO-1 and SAO-2 provide concentration outputs with small and large phase shifts, respectively, against temperature oscillation inputs; SAO-4 and SAI-3 provide frequency-doubled outputs. The resonance phenomenon can be regarded as a selective output of concentration oscillations against a small change in thermal input.

To summarize Section 2, stable concentration oscillations are described, in which repeated cycles periodically provide the same states at the same temperatures with regard to concentration, direction of reaction, and reaction rate. Stable concentration oscillations are classified according to their waveforms in concentration/time profiles, the shapes of hysteresis curves in concentration/temperature profiles, the nature of self-catalytic reactions, and their relationships with equilibrium (Figure 4). In terms of their relationships with equilibrium, SAO-1, SAO-2, SAO-3, and SAO-4 occur involving equilibrium overlapping, in which equilibria are achieved and memory of previous cycles is lost; SAI-1, SAI-2, SAI-3, and SAI-4 occur involving equilibrium intersecting, in which the balanced states of reactions and temperature change are achieved; SAN-1 and SAN-2 occur involving equilibrium noncontact and the balanced states.

## 3. Unstable Concentration Oscillations

### 3.1. Unstable and Stable Concentration Oscillations

**Figure 5:**
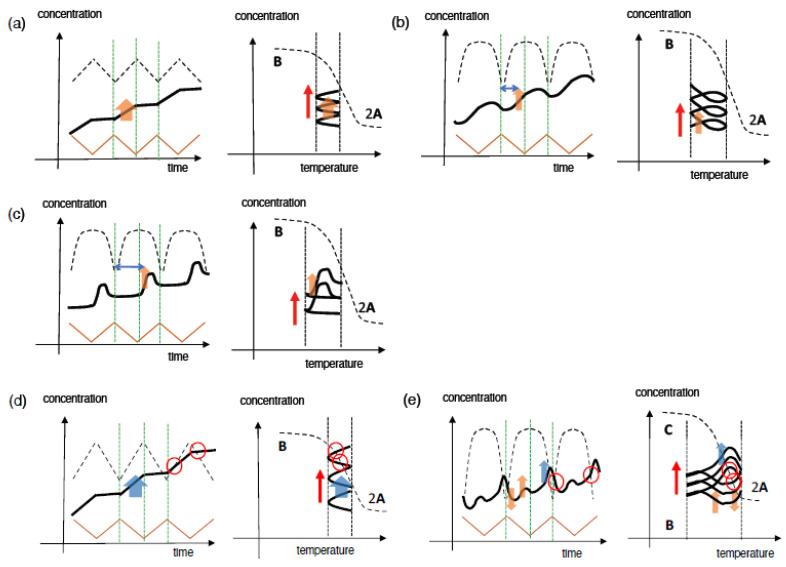
Classification of unstable concentration oscillations involving 2**A** + **B**→2**B** and/or 2**A** + **C**→2**C**. Unstable concentration oscillations with amplify hysteresis involving equilibrium noncontact: (**a**) wrinkled concentration oscillation with zigzag hysteresis (UAN-1), (**b**) gradient sinusoidal concentration oscillation with loop hysteresis (UAN-2), and (**c**) gradient retarded concentration oscillation with swing hysteresis (UAN-3). Unstable concentration oscillations with amplify hysteresis involving equilibrium sliding: (**d**) wrinkled concentration oscillation with zigzag hysteresis (UAI-1) and (**e**) gradient frequency-doubled concentration oscillation with twist loop hysteresis (UAI-2). Orange lines indicate temperature oscillations; red arrows indicate the direction of unstable concentration oscillations; orange arrows indicate self-catalytic reactions; blue bold arrows in (**d**,**e**) indicate self-catalytic reactions with equilibrium sliding; red circles indicate intersections of equilibrium curves; dashed black lines indicate equilibrium curves; blue arrows in (**b**,**c**) indicate phase shifts; green vertical dashed lines indicate switching between cooling and heating and are provided to show difference in phase.

In Section 2, we have described stable concentration oscillations with delay and amplify hysteresis (Figure 4). Temperature oscillations provide periodic concentration oscillations with the same amplitude, waveform, period, and frequency in repeated cycles, which result in the same state at the same temperature in terms of concentration, direction of reactions, and reaction rate. They occur involving equilibrium or under the balanced state of reactions and temperature change. In Section 3, we describe unstable concentration oscillations with amplify hysteresis involving self-catalytic reactions (Figure 5). Temperature oscillations provide quasiperiodic concentration oscillations with different amplitudes, waveforms, periods, or frequencies in repeated cycles, which result in the different states at the same temperature in terms of concentration, direction of reactions, and reaction rate. They occur under the disrupted balanced state of reactions and temperature change. An unstable concentration oscillation is transformed into a stable concentration oscillation after a sufficient number of cycles, during which various transient phenomena appear.

### 3.2. Unstable Concentration Oscillations with Amplify Hysteresis

Unstable concentration oscillations with amplify hysteresis are classified according to their waveforms in concentration/time profiles, the shapes of hysteresis curves in concentration/temperature profiles, the nature of self-catalytic reactions, and their relationships with equilibrium (Figure 5).

Consider the reaction 2**A**
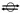
**B** involving 2**A** + **B**→2**B** with **B** thermodynamically stable at low temperatures and 2**A** at high temperatures. When an unstable concentration oscillation shows a continuous increase in [**B**] with different increments during cooling and heating, repeated cycles provide small wrinkles in the concentration/temperature profile (Figure 5a). The wrinkled concentration oscillation is accompanied by a zigzag hysteresis, which is named UAN-1. UAN-1 appears owing to a strong 2**A** + **B**→2**B**, which occurs during both cooling and heating (Figure 5a, orange arrows). It is an interesting observation that [**B**] increases during heating, despite the decrease in thermodynamic stability of **B** upon heating.

An unstable sinusoidal concentration oscillation showing a gradual increase in the concentration occurs, in which [**B**] increases during cooling owing to 2**A** + **B**→2**B** and decreases during heating (Figure 5b). The gradient sinusoidal concentration oscillation provides the loop hysteresis, in which the cooling and heating curves cross, which is named UAN-2. In contrast to UAN-1, which involves a strong 2**A** + **B**→2**B** with an increase in [**B**] during both cooling and heating (Figure 5a), UAN-2 shows an increase in [**B**] only during cooling.

Another unstable concentration oscillation showing gradual changes of retarded concentration oscillation occurs, in which [**B**] increases during heating but not during cooling owing to a significant delay of 2**A** + **B**→2**B** (Figure 5c, orange arrows). The gradient retarded concentration oscillation with swing hysteresis is named UAN-3. UAN-3 is different from UAN-2 (Figure 5b, orange arrows), in which [**B**] increases during cooling. The larger phase shift in UAN-3 is compared with a smaller phase shift in UAN-2 (Figure 5b,c, blue and orange arrows), which is shown by the increase in [**B**] owing to 2**A** + **B**→2**B** on the left and right sides of the central dashed vertical green lines, respectively.

The above unstable concentration oscillations occur out of equilibrium without contact with the equilibrium curves (Figure 5a–c). Unstable concentration oscillations can also occur through equilibrium intersecting repeated many times, which is named equilibrium sliding (Figure 5d,e). It is due to self-catalytic reactions along the equilibrium curve (Figure 5d,e, blue arrows). Different types of equilibrium sliding occurs depending on the nature of self-catalytic reactions. Wrinkled concentration oscillation with zigzag hysteresis occurs owing to a strong 2**A** + **B**→2**B**, which is named UAI-1 (Figure 5d). Gradient retarded concentration oscillation with twisted loop hysteresis occurs owing to the competition between 2**A** + **B**→2**B** and 2**A** + **C**→2**C**, which is named UAI-2 (Figure 5e).

### 3.3. Transformation from Unstable Concentration Oscillations to Stable Ones

**Figure 6:**
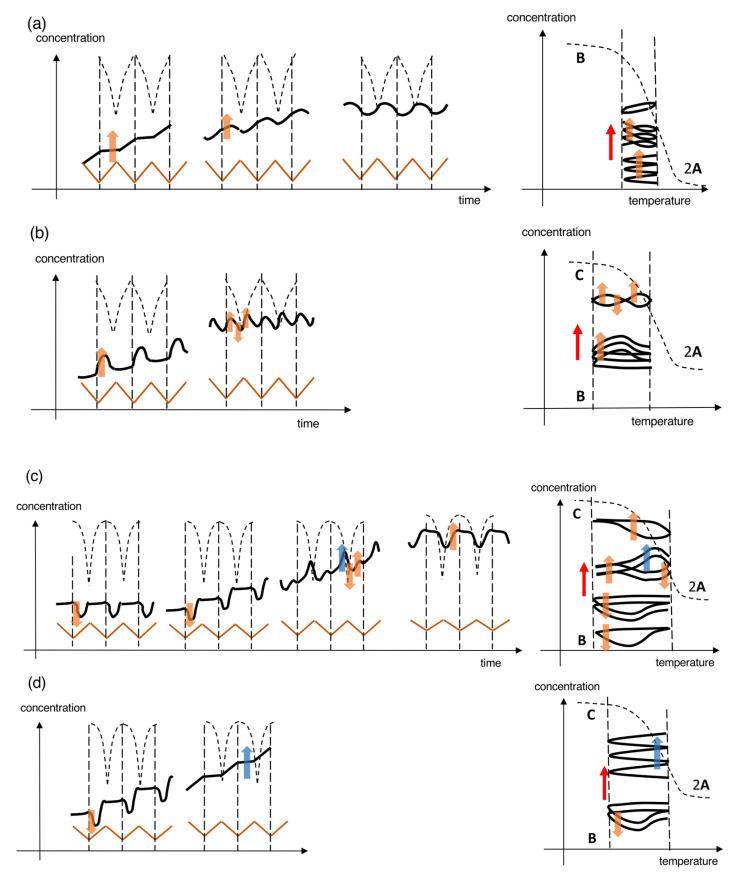
Examples of transformations from unstable to stable concentration oscillations involving competitive 2**A** + **B**→2**B** and/or 2**A** + **C**→2**C**: (**a**) UAN-1 to UAN-2 to SAI-4, (**b**) UAN-3 to SAI-3, (**c**) SAN-1 to UAN-3 to UAI-2 to SAI-1, and (**d**) UAN-3 to UAI-1. Orange bold arrows indicate self-catalytic reactions; red arrows indicate transformation from unstable concentration oscillations to stable ones; blue arrows indicate self-catalytic reactions with equilibrium sliding; dashed lines indicate equilibrium; dashed vertical black lines indicate switching between cooling and heating and are provided to show difference in phase.

An unstable concentration oscillation is transformed into a stable concentration oscillation after a sufficient number of cycles, resulting in balanced states (Figure 5). Unstable concentration oscillations occur out of equilibrium and generally proceed in the direction towards equilibrium, during which complex transient phenomena occur by self-catalytic reactions. The resulting stable concentration oscillation can involve equilibrium intersecting and equilibrium noncontact. The transformations, which have been obtained in our study, are analyzed by models based on the classifications in Figure 4 and Figure 5 (Figure 6). The transformations involving a single self-catalytic reaction 2**A** + **B**→2**B** and two competitive self-catalytic reactions 2**A** + **B**→2**B** and 2**A** + **C**→2**C** are described in Section 3.3.1 and Section 3.3.2, respectively.

#### 3.3.1. A model with a Single Self-Catalytic Reaction

UAN-1 is transformed to UAN-2 and then to SAI-4. The transformation involves the reduction in the strength of 2**A** + **B**→2**B** (Figure 6a). The strength of 2**A** + **B**→2**B** in UAN-1, which occurs during both cooling and heating, is reduced in UAN-2, which occurs only during cooling. Then, UAN-2 is transformed to SAI-4 with a minimal 2**A** + **B**→2**B**. A direct transformation from UAN-1 to SAN-4 can also occur.

#### 3.3.2. Models with Two Competitive self-Catalytic Reactions

Complex phenomena can occur during the transformations from unstable concentration oscillations to stable ones, when competition of 2**A** + **B**→2**B** and 2**A** + **C**→2**C** are involved. For example, equilibrium sliding occurs involving equilibrium intersecting repeated many times; an unstable concentration oscillation proceeds in a direction away from equilibrium; a stable concentration oscillation is transformed to an unstable concentration oscillation.

UAN-3 may be transformed into SAI-3, which exhibits frequency-doubled concentration oscillation (Figure 6b). The transformation involves the switching from a single 2**A** + **B**→2**B** to two competitive 2**A** + **B**→2**B** and 2**A** + **C**→2**C**, (Figure 6b, orange arrows). Of note is that 2**A** + **B**→2**B** proceeds in the direction away from equilibrium, and 2**A** + **C**→2**C** proceeds in the direction towards equilibrium, in which **C** is thermodynamically more stable than **B**.

SAN-1 may be transformed to UAN-3 to UAI-2 and finally to SAI-1 (Figure 6c). A stable concentration SAN-1 involving 2**A** + **B**→2**B** is transformed to an unstable concentration oscillation UAN-3, which is a notable phenomenon reverse to the transformation from an unstable concentration oscillation to a stable one. Then, UAN-3 is transformed to UAI-2 because of the involvement of competitive 2**A** + **B**→2**B** and 2**A** + **C**→2**C**, which exhibits frequency-doubled concentration oscillation. Equilibrium sliding also occurs in UAI-2 owing to 2**A** + **C**→2**C** occurring along the equilibrium curve (Figure 6c, blue arrow). Eventually, UAI-2 is transformed to SAI-1, which involves 2**A** + **C**→2**C**. The overall transformation process involves the switching from 2**A** + **B**→2**B** to 2**A** + **C**→2**C**.

UAN-3 involving gradient retarded concentration oscillation with swing hysteresis may be transformed to UAI-1 involving wrinkled concentration oscillation with zigzag hysteresis (Figure 6d), which shows equilibrium sliding (Figure 6d, blue arrow). It is noted that UAN-3 involving 2**A** + **B**→2**B** is transformed to UAI-1 involving a strong 2**A** + **C**→2**C**.

The three examples of the transformations in Figure 6b–d occur by the switching from 2**A** + **B**→2**B** to 2**A** + **C**→2**C**, and the switching phenomena can be described by the change of domains from 2**A** + **B**→2**B** to 2**A** + **C**→2**C** in concentration/temperature profiles, as will be described in Section 3.4.

It is interesting to determine whether thermal history of unstable concentration oscillations affects the resulting stable concentration oscillations. It can be shown by analysis of whether different unstable concentration oscillations are transformed into the same or different stable ones. Examples showing the transformations to the same ones will be described in Section 4 and Section 6.1.2. The general rule on history dependence is a subject in future investigation.

To summarize Section 3.1, Section 3.2 and Section 3.3, diverse unstable concentration oscillations occur when the balance between reactions and temperature change is disrupted, which are classified according to their waveforms in concentration/time profiles, the shapes of hysteresis curves in concentration/temperature profiles, the nature of self-catalytic reactions, and their relationships with equilibrium. The transformation from unstable concentration oscillations to stable ones are described on the basis of their classification, and experimental examples will be described in Section 5 and Section 6. The precise mechanisms underlying the balanced states and disrupted balanced states in stable and unstable concentration oscillations, respectively, are not clear at present, and theoretical studies are another subject in the future, in which the concept of limit cycles may provide a clue [44].

### 3.4. Domains of Self-Catalytic Reactions in Concentration/Temperature Profiles

**Figure 7:**
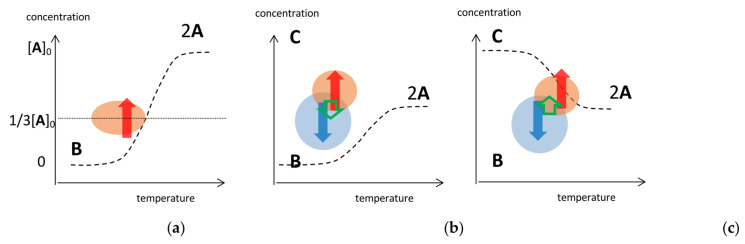
Domains of self-catalytic reactions in a system of (**a**) a single self-catalytic reaction to form **B** and (**b**,**c**) that of two competitive self-catalytic reactions to form **B** and **C**. Self-catalytic reaction domains can be (**b**) distant from or (**c**) along the equilibrium curve (**c**). Blue and red arrows indicate self-catalytic reactions; blue and red circles indicate self-catalytic reaction domains of 2**A** + **B**→2**B** and 2**A** + **C**→2**C**, respectively; common domains of blue and red indicate the competition between the self-catalytic reactions; dashed lines indicate equilibrium; green arrows indicate switching the domains of 2**A** + **B**→2**B** to 2**A** + **C**→2**C**.

A rough sketch on the strength of self-catalytic reactions can be described by domains in concentration/temperature profiles, which shows the nature of stable and unstable concentration oscillations with amplify hysteresis. UAN-1 and UAI-1 involving wrinkle concentration oscillations with zigzag hysteresis occur in the domain of a strong 2**A** + **B**→2**B** (Figure 7a, red circle). Of note is that the rate of 2**A** + **B**→2**B** starting from [**A**] = [**A**]_0_ is largest at approximately [**A**] = 1/3[**A**]_0_ [30]. When 2**A** + **B**→2**B** and 2**A** + **C**→2**C** compete, UAN-2 can occur in the domains of 2**A** + **B**→2**B** (Figure 7b, blue circle) and UAN-3 in the domain of 2**A** + **C**→2**C** (Figure 7b, red circle). SAI-3 and UAI-2 involving frequency-doubled concentration oscillations appear at the common domain of blue and red circles owing to their competition. When 2**A** + **C**→2**C** occurs along the equilibrium curve, UAI-1 appears involving equilibrium sliding (Figure 7c, red circle). Transformations from unstable concentration oscillations to stable ones show a general tendency of switching in the domains from 2**A** + **B**→2**B** to 2**A** + **C**→2**C** by repeated cycles, because of the approach toward the equilibrium (Figure 7b,c, green arrows).

Experimental results on stable and unstable concentration oscillations in Section 4, Section 5 and Section 6 are described employing Δε as an equivalent of concentration; Δε is obtained by circular dichroism (CD) spectroscopy, which is highly sensitive to conformational changes of helicene oligomers, which exhibit extremely large absolute values. When 2**A**
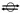
**B** with two species **A** and **B** are involved, [**B**] and Δε are proportional, Δε = (2Δe**_B_**[**B**] + Δe**_A_**[**A**])/[**A**]_0_≒2Δe**_B_**[**B**]/[**A**]_0_ and [**A**]_0_ = [**A**] + 2[**B**], because Δε**_A_**≒0 cm^−1^ M^−1^ in the experiments of this study, in which Δε**_A_** and Δε**_B_** are the Δε of pure **A** and **A**, respectively, and [**A**]_0_ is the total [**A**]. In contrast, a system of competitive 2**A**
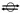
**B** and 2**A**
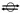
**C** with three species **A**, **B**, and **C** provide Δε = {2Δε**_B_**[**B**] + 2Δε**_C_**[**C**] + Δε**_A_**[**A**]}/[**A**]_0_≒2Δε**_B_**([**B**] − [**C**])/[**A**]_0_ and [**A**]_0_ = [**A**] + 2[**B**] + 2[**C**], because Δε**_B_**≒−Δε**_C_** and Δε**_A_**≒0 cm^−1^ M^−1^ in the experiments of this study, in which Δε**_C_** is the Δε of pure **C**. Then, Δε is proportional to [**B**] − [**C**]. For the determination of [**A**], [**B**], and [**C**], UV-vis data can be employed, by which ε = {2ε**_B_**([**B**] + [**C**]) + ε**_A_**[**A**]}/[**A**]_0_ is provided because ε**_B_**≒ε**_C_**, where ε, ε**_A_**, ε**_B_**, and ε**_C_** are the molar absorption coefficients of the sample, pure **A**, **B**, and **C**, respectively [45]. Concentration oscillations can conveniently be discussed with Δε as an equivalent of concentration using Δε/time and Δε/temperature profiles.

### 3.5. Scope of This Article

Daily and yearly temperature oscillations can affect behaviors of living things, which employ the conversion of environmental temperature oscillations into concentration oscillations by chemical reactions. To control such biological events, it is critical to understand the effect of temperature oscillations on chemical reactions, especially in the presence of amplification. Such a study has not previously been conducted in a systematic manner. This article attempts to discuss the effect of temperature oscillations on chemical reactions involving self-catalytic reactions, which is based on the classifications of concentration oscillations. Results obtained in our previous works are organized from a different perspective and are comparatively discussed with regard to stable and unstable concentration oscillations with amplify hysteresis.

**Figure 8:**
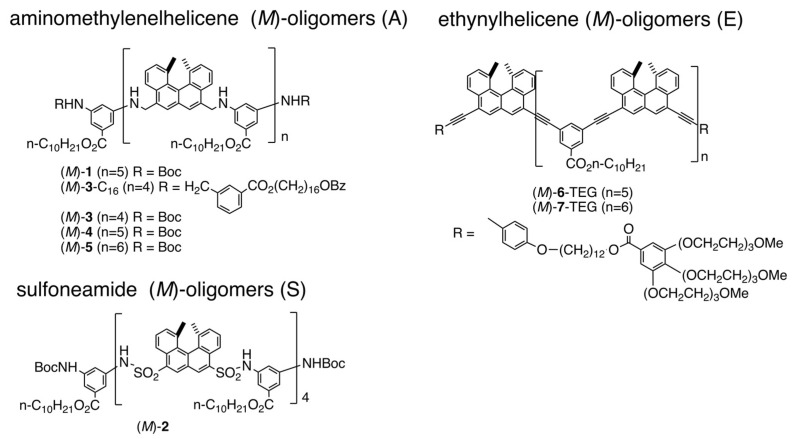
Chemical structures of helicene oligomers.

Helicenes are an interesting group of compounds with distorted helical aromatic ring systems [46,47,48,49,50,51,52]. We have developed helicene oligomers, which form homo- and hetero-double-helices in solution (Figure 8) [27,29,30,31,32]. Heating and cooling induce reversible association and dissociation reactions, which exhibit amplify hysteresis involving self-catalytic reactions. Aminomethylene, sulfonamide, and ethynyl helicene oligomers described in this article are indicated by (A), (S), and (E), respectively.

Classifications of stable and unstable concentration oscillations are summarized below (Table 1).

## 4. Stable Concentration Oscillations with Delay Hysteresis

Aminomethylene (*M*)–pentamer (*M*)–**1** exhibits stable concentration oscillations SD-1 and SD-2 with delay hysteresis caused by the reaction 2**A**_1_(A)
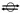
**B**_1_(A) between homo-double-helix **B**_1_(A) with a strong negative Δε at 310 nm and random-coil 2**A**_1_(A) with Δε 0 cm^−1^ M^−1^ [35]. Amplification through self-catalytic reaction is not involved for 2**A**_1_(A)
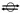
**B**_1_(A). Δε is used to show the concentration of **B**_1_(A) in the following discussions, as noted in Section 3.4.

**Figure 9:**
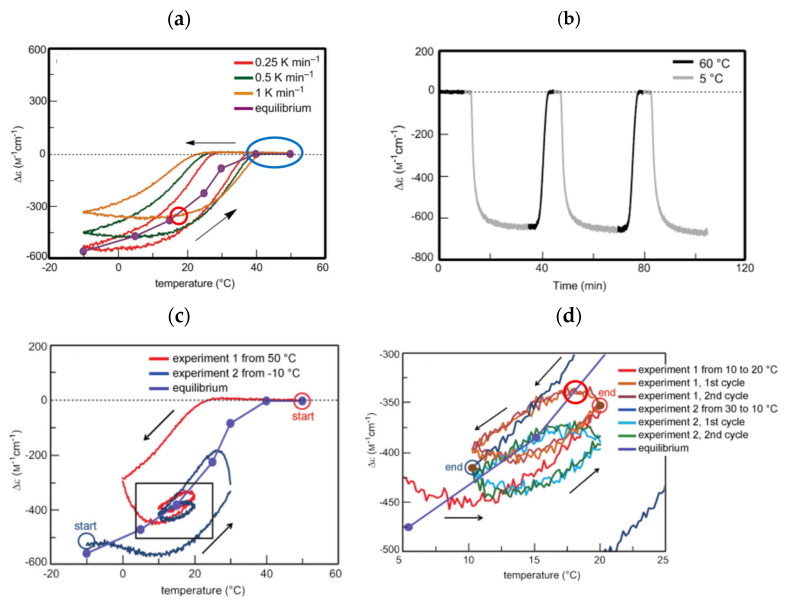
SD-1 of (*M*)–**1** in toluene (0.5 mM) induced by (**a**) a 50/−5 °C temperature oscillation, which is shown by Δε/temperature profile and (**b**) a square temperature oscillation between 60 and 5 °C, which is shown by Δε/time profile. (**c**) SD-2 shown by a 50/−5 °C temperature oscillation at a rate of 0.15 K min^−1^ starting from 50 and −5 °C. (**d**) An expansion of (**c**). Blue circle in (**a**) indicates equilibrium overlapping; red circles in (**a**,**d**) indicate equilibrium intersecting. Reproduced from ref. [35] with permission from Wiley.

SD-1 is shown by Δε/temperature profile (Figure 9). (*M*)–**1** in toluene (0.5 mM) at 50 °C with fully dissociated 2**A**_1_(A) was cooled to −10 °C and heated to 50 °C at a rate of 0.25 K min^−1^. Δε 0 cm^−1^ M^−1^ at 50 °C decreased to −530 cm^−1^ M^−1^ at −10 °C, which indicated the formation of **B**_1_(A) (Figure 9a, red line). Heating to 50°C induced the dissociation to 2**A**_1_(A), which increased to Δε 0 cm^−1^ M^−1^. Different cooling and heating curves appear on both sides of the equilibrium curve with equilibrium overlapping at high temperatures (Figure 9a, blue circle), which provide a normal hysteresis owing to the delay of 2**A**_1_(A)
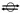
**B**_1_(A) versus the temperature oscillation. Equilibrium at high temperatures is achieved owing to the equilibrium overlapping and provides the stable concentration oscillation SD-1. The stable nature is confirmed by the appearance of the same cycles in the Δε/time profile, in which a square temperature oscillation between 60 and 5 °C is provided (Figure 9b). When the rate of temperature change is increased to 1.0 K min^−1^, Δε reached at −5 °C increases, and equilibrium intersecting occurs during heating (Figure 9a, orange line and red circle).

SD-2 occurs under a temperature oscillation over a narrow temperature range (Figure 3b). When 2**A**_1_(A) was cooled from 50 to 0 °C followed by the temperature oscillation between 20 and 10 °C at a rate of 0.15 K min^−1^, SD-2 with oval hysteresis occurred at Δε close to −400 cm^−1^ M^−1^ involving equilibrium intersecting (Figure 9c,d, red circle). A different thermal history of the temperature oscillation was provided starting from −10 °C containing **B**_1_(A), and the mixture was heated to 30 °C followed by a 20/10 °C temperature oscillation at a rate of 0.15 K min^−1^. Two temperature oscillations starting from 50 and −10 °C provide SD-2 close to each other, which indicates a small effect of their thermal history (Section 3.3).

## 5. Unstable Concentration Oscillations with Amplify Hysteresis Involving a Single Self-Catalytic Reaction

### 5.1. Stable Concentration Oscillations Involving Equilibrium Overlapping

Concentration oscillations with amplify hysteresis of sulfonamide (*P*)-tetramer (*P*)-**2** caused by 2**A**(S)
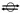
**B**(S) involving a single self-catalytic reaction 2**A**(S) + **B**(S)→2**B**(S) are discussed in Section 5.1, in which homo-double-helix **B**(S) with the strong positive Δε at 320 nm and random-coil 2**A**(S) with the negative Δε are interconverted [53]. Behavior of stable concentration oscillations of (*P*)-**2** is complex compared to SD-1 and SD-2 of (*M*)-**1** shown in Section 4. A transformation from an unstable concentration oscillation to a stable one, that from UAN-1 to SAN-2, is also described at different concentrations and temperature ranges.

**Figure 10:**
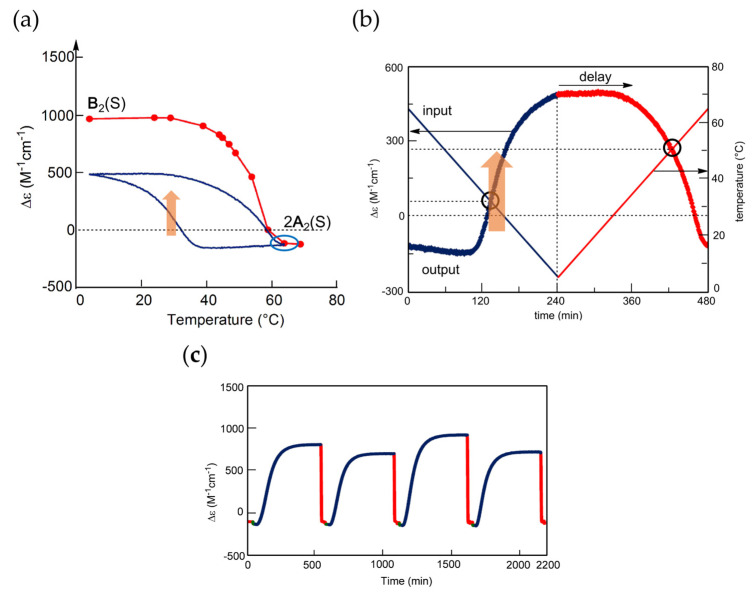
SAO-1 of (*P*)-**2** by a 65/5 °C temperature oscillation in 1,3–difluorobenzene (0.5 mM) at a rate of 0.25 K min^−1^ shown by (**a**) Δε/temperature and (**b**) Δε/time profiles. (**c**) A stable concentration oscillation provided by a square temperature oscillation between 70, 50, and 45 °C. Blue circle in (**a**) indicates equilibrium overlapping; orange bold arrows indicate self-catalytic reaction. Reproduced from ref. [53] with permission from Wiley.

Stable concentration oscillation SAO-1 occurs involving equilibrium overlapping at high temperatures (Figure 4a). (*P*)-**2** in fluorobenzene (0.5 mM) at 65 °C containing fully dissociated 2**A**(S) exhibiting Δε −140 cm^−1^ M^−1^ was cooled and heated between 65 and 5 °C at a rate of 0.25 K min^−1^ (Figure 10a). Upon cooling from 65 to 40 °C, the cooling curve in the Δε/temperature profile moved away from the equilibrium curve without change in Δε, which is caused by the delay of 2**A**(S) + **B**(S)→2**B**(S). At 40 °C, Δε started to increase, owing to 2**A**(S) + **B**(S)→2**B**(S), and reached +490 cm^−1^ M^−1^ at 5 °C, which is a metastable state formed by retadation of the reaction upon cooling. Δε decreased upon heating from 5 °C and reached −140 cm^−1^ M^−1^ at 65 °C. Different sigmoidal curves are obtained upon cooling and heating exhibiting a seminormal hysteresis, and the cooling and heating curves appear far below the equilibrium curve. It is in contrast to SD-1 of (*M*)–**1**, hysteresis curves of which appear at close to the equilibrium curve on both sides (Figure 9a). Equilibrium overlapping of (*P*)-**2** at high temperatures provides SAO-1 (Figure 10a, blue circle), which shows a sinusoidal concentration oscillation with phase shift versus temperature oscillation (Figure 10b). The stable nature is confirmed by an experiment of a square temperature oscillation between 70, 50, and 45 °C, providing essentially the same waveform in every cycle (Figure 10c).

### 5.2. Transformations from Unstable Concentration Oscillations to Stable Ones

**Figure 11:**
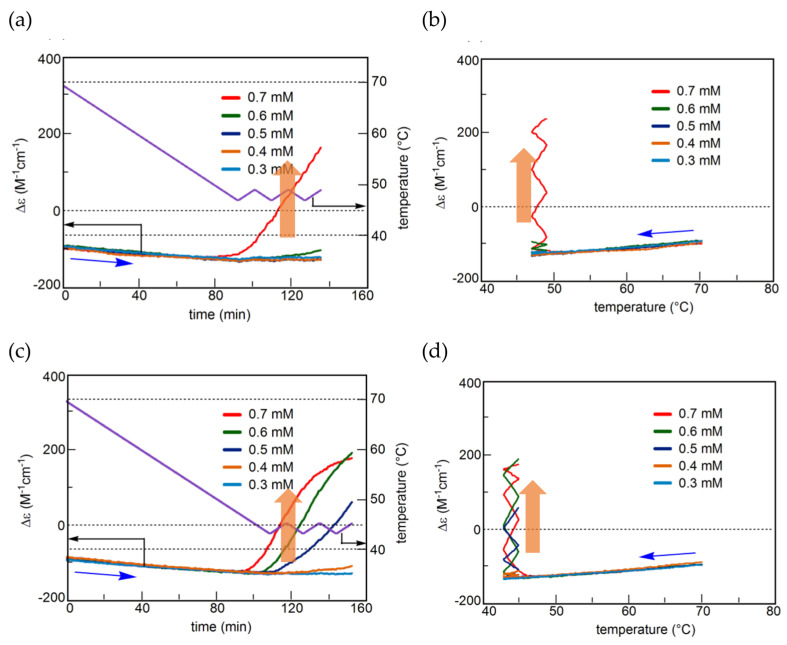
UAN-1 of (*P*)–**2** in 1,3–difluorobenzene at a rate of 0.25 K min^−1^ shown by Δε/time and Δε/temperature profiles, which are induced by (**a**,**b**) 49/47 °C temperature oscillations and (**c**,**d**) 45/43 °C temperature oscillations. Reproduced from ref. [54] with permission from Wiley.

Unstable concentration oscillations UAN-1, which lack equilibrium overlapping, occur by temperature oscillations over narrow temperature ranges (Figure 5a and Figure 11) [54]. (*P*)-**2** in 1,3-difluorobenzene (0.7 mM) was heated to 70 °C to form fully dissociated 2**A**(S) under equilibrium, cooled to 47 °C at a rate of 0.25 K min^−1^, and provided with a temperature oscillation between 49 and 47 °C. Such a temperature oscillation is noted as 49/47 °C temperature oscillation hereafter. Continuous increases in Δε both upon cooling and heating occur in the direction towards the formation of thermodynamically stable **B**(S) (Figure 11a, red line). Thus, UAN-1 with wrinkled concentration oscillation and zigzag hysteresis appears owing to a strong 2**A**(S) + **B**(S)→2**B**(S) (Figure 11b, red line). Once initiated, 2**A**(S) + **B**(S)→2**B**(S) significantly increases **B**(S), despite the decrease in the thermodynamic stability of **B**(S) upon heating (Figure 11, orange bold arrows).

UAN-1 appears at 0.7 mM, and essentially no reaction occurs upon cooling and heating at the concentrations of 0.6 mM and below (Figure 11a,b). This is a concentration threshold phenomenon, in which 2**A**(S) + **B**(S)→2**B**(S) occurs by a small increase in the concentration from 0.6 to 0.7 mM. The threshold concentration is affected by temperature range of the temperature oscillation, and a 45/43 °C temperature oscillation provides UAN-1 at 0.5 mM and not at 0.4 mM (Figure 11c,d). A small difference in the temperature range between 49/47 and 45/43 °C temperature oscillations by 4 °C provides different threshold concentrations of 0.6 and 0.4 mM, respectively. Note that UAN-1 occurs far away from the equilibrium curve, in which the equilibrium states are described by Δε approximately +460 cm^−1^ M^−1^ (55 °C), +670 cm^−1^ M^−1^ (50 °C), and +800 cm^−1^ M^−1^ (45 °C) in 0.5 mM solution [53].

**Figure 12:**
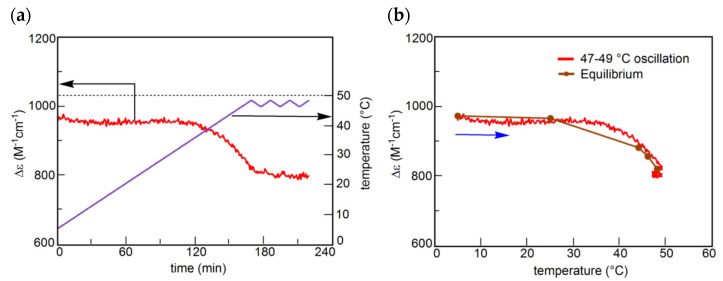
SE-2 by a 49/47 °C temperature oscillation of (*P*)–**2** in 1,3–difluorobenzene (0.7 mM) at a rate of 0.25 K min^−1^ shown by (**a**) Δε/time and (**b**) Δε/temperature profiles. Orange line in (**b**) is equilibrium curve. Reproduced from ref. [54] with permission from Wiley.

UAN-1, which occurs out of equilibrium, is compared with SE-2, which occurs under equilibrium (Figure 11 and Figure 12) [54]. A solution (0.7 mM) of **B**(S) with Δε approximately +950 cm^−1^ M^−1^ under equilibrium at 5 °C is heated to 49 °C at a rate of 0.25 K min^−1^, which provides another equilibrium state with Δε approximately +800 cm^−1^ M^−1^. When a 49/47 °C temperature oscillation is provided, a very small change of Δε appears (Figure 12a, red line) along the equilibrium curve (Figure 12b, red and orange lines). The very small change is in contrast to the significant concentration oscillation of UAN-1 induced by the same temperature oscillation (Figure 11a,b, red lines), which involves the amplification through 2**A**(S) + **B**(S)→2**B**(S).

**Figure 13:**
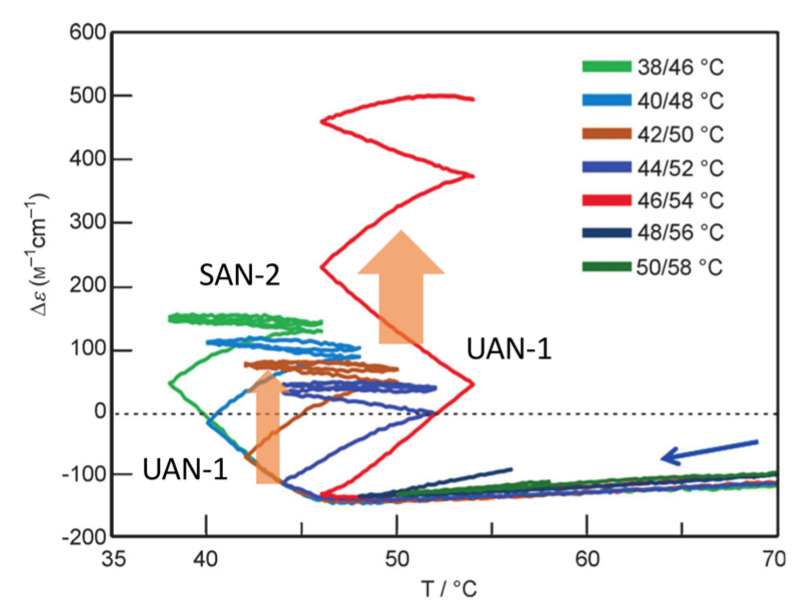
Transformations from UAN-1 to SAN-2 of (*P*)–**2** in 1,3–difluorobenzene (0.7 mM) by temperature oscillations with different temperature ranges at a rate of 0.25 K min^−1^. Orange arrows indicate self-catalytic reactions. Reproduced from ref. [54] with permission from Wiley.

Changes in the temperature range of temperature oscillations substantially affect concentration oscillations. When 58/50 °C and 56/48 °C temperature oscillations are provided at a rate of 0.25 K min^−1^, essentially no reaction occurs by appearance of the same cooling and heating curves (Figure 13, green and blue lines) [54]. In contrast, a 54/46 °C temperature oscillation provides UAN-1 reaching Δε approximately +500 cm^−1^ M^−1^ at the third heating (Figure 13, red line). The difference of the temperature range by 2 °C exhibits quite different concentration oscillations, which is a temperature threshold phenomenon.

Transformations from UAN-1 to SAN-2 occur, when 52/44, 50/42, 48/40, and 46/38 °C temperature oscillations are provided (Figure 6a and Figure 13). Significant increases in Δε in the UAN-1 appear in the first cycles, and the second and subsequent cycles provide SAN-2 with small hysteresis areas. The transformations occur with the reduction of the intensity of 2**A**(S) + **B**(S)→2**B**(S) from strong ones in UAN-1 to minimal ones in SAN-2. When lower temperature ranges of the temperature oscillations are provided, the SAN-2 occur with increased Δε ranges providing different balanced states between reactions and temperature change.

A resonance phenomenon appears (Section 2.3.5), in which significantly different concentration oscillations occur by changing the temperature range (Figure 13): No reaction occurs by a 58/50 °C temperature oscillation; UAN-1 occurs by a 54/46 °C temperature oscillation; transformations from UAN-1 to SAN-2 occur by 52/44, 50/42, 48/40, and 46/38 °C temperature oscillations. The different concentration oscillations occur owing to the strong 2**A**(S) + **B**(S)→2**B**(S) induced by the 54/46 °C temperature oscillation and to the reduced intensities at the temperature ranges above and below. Additionally, 2**A**(S) + **B**(S)→2**B**(S) shows a high sensitivity against a small change in the temperature range.

### 5.3. Effect of Temperature Change Rate

**Figure 14:**
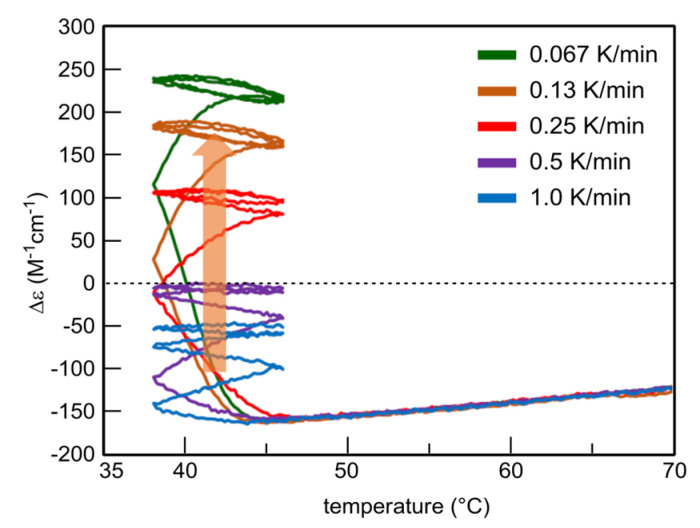
Transformations from UAN-1 to SAN-2 of (*P*)–**2** in 1,3–difluorobenzene (0.5 mM) by a 46/38 °C temperature oscillation at different rates. Orange arrow indicates self-catalytic reaction. Reproduced from ref. [55] with permission from Wiley.

UAN-1 is transformed into different SAN-2 under a 46/38 °C temperature oscillation depending on the temperature change rates between 0.067 and 1.0 K min^−1^ (Figure 14) [55]. SAN-2 at a rate of 0.067 K min^−1^ occurs between Δε +200 and +250 cm^−1^ M^−1^; SAN-2 at a rate of 1.0 K min^−1^ occurs close to −50 cm^−1^ M^−1^. These SAN-2 exhibit a gradual increase in Δε range by the decrease in the rate, because strong 2**A**(S) + **B**(S)→2**B**(S) induces larger increases in Δε under the lower rates. Thus, balanced states between reactions and temperature change are affected by temperature change rate as well as temperature range.

### 5.4. Higher Order Stable Concentration Oscillation

**Figure 15:**
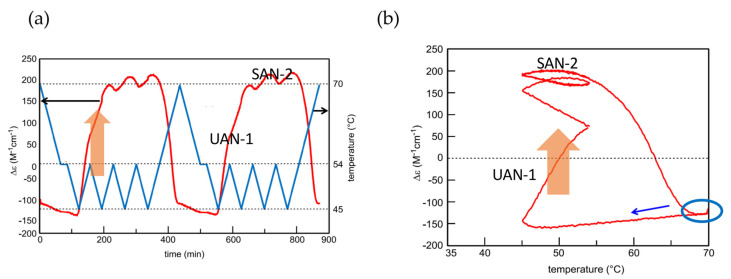
A higher order stable concentration oscillation of (*P*)–**2** in 1,3–difluorobenzene (0.5 mM) at a rate of 0.25 K min^−1^ by (**a**) a 54/45 °C temperature oscillation and heating to 70 °C, which is shown by Δε/time and (**b**) Δε/temperature profiles. Blue circle indicates equilibrium overlapping; orange bold arrows indicate self-catalytic reaction. Reproduced from ref. [55] with permission from Wiley.

The above experiments provide temperature oscillations with periodic cooling and heating cycles. Of note is that a cycle can involve a set of different temperature oscillations, called pseudo-periodic temperature oscillations, which can provide a higher order stable concentration oscillation (Figure 15a) [55]. A transformation from UAN-1 to SAN-2 occurs by a 54/42 °C temperature oscillation at a rate of 0.25 K min^−1^. Then, heating to 70 °C forms 2**A**(S) with Δε approximately −120 cm^−1^ M^−1^, which is accompanied by equilibrium overlapping at above 68 °C (Figure 15b, blue circle). A subsequent cycle of the pseudo-periodic temperature oscillation with a set of a 54/42 °C temperature oscillation and heating to 70 °C provides essentially the same cycle of Δε (Figure 15a). This is a higher order stable concentration oscillation involving UAN-1, SAN-2, and formation of 2**A**(S) by self–catalytic reaction (Figure 15b, organe bold arrow).

### 5.5. Domains of Self-Catalytic Reaction of (P)-2

**Figure 16:**
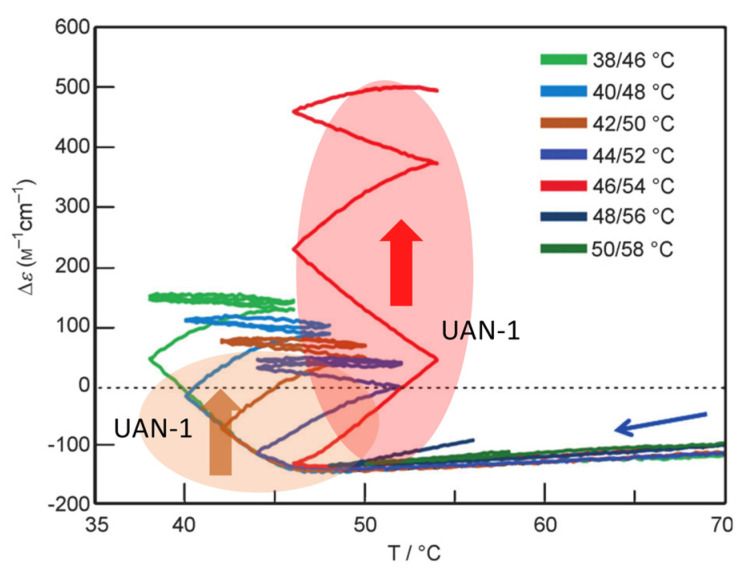
Domains of a self-catalytic reaction of (*P*)-**2** in 1,3–difluorobenzene (0.7 mM) at a rate of 0.25 K min^−1^. Red and orange circles indicate strong 2**A**(S) + **B**(S)→2**B**(S) domains, and red and orange arrows indicate their directions. Figure 13 is reproduced to compare with cooling and heating curves. Reproduced from ref. [54] with permission from Wiley.

2**A**(S) + **B**(S)→2**B**(S) in 1,3-difluorobenzene (0.7 mM) can be described by domains in a Δε/temperature profile (Figure 7 and Figure 16). A domain of a strong 2**A**(S) + **B**(S)→2**B**(S) appears between 45 and 55 °C in the range of Δε between −100 and +500 cm^−1^ M^−1^ (Figure 16, red arrow and circle), which exhibits UAN-1 (Figure 13). The observation is consistent with 2**A**(S) + **B**(S)→2**B**(S) exhibiting a maximum rate at approximately [**B**(S)] = 1/3[**A**(S)]_0_ [29], which is in the range of Δε +200 to +300 cm^−1^ M^−1^ in this system. Another domain of a strong 2**A**(S) + **B**(S)→2**B**(S) appears between 35 and 50 °C in the range of Δε between −100 and +50 cm^−1^ M^−1^, which also exhibits UAN-1 (Figure 16, orange arrow and circle).

## 6. Unstable Concentration Oscillations with Amplify Hysteresis Involving Two Competitive Self-Catalytic Reactions

Unstable concentration oscillations with amplify hysteresis exhibit complex phenomena, when temperature oscillations are provided to a reaction system of 2**A**(A)
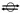
**B**(A) and 2**A**(A)
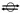
**C**(A) involving two competitive self-catalytic reactions 2**A**(A) + **B**(A)→2**B**(A) and 2**A**(A) + **C**(A)→2**C**(A). Mixtures of aminomethylene (*P*)- and (*M*)-oligomers form random-coil 2**A**(A) at high temperatures and hetero-double helices **B**(A) and **C**(A) at low temperatures with the enantiomeric helical senses [56,57]. Unstable concentration oscillations and their transformations into stable concentration oscillations are described by Δε/time and Δε/temperature profiles, in which Δε is proportional to [**B**(A)] − [**C**(A)] (Section 3.4). The property of concentration oscillations varies by oligomer structures, which include the numbers of the helicene unit and the functional groups at the terminal positions. Section 6.1 describes a combination of (*M*)–pentamer (*M*)-**4** and (*P*)–tetramer (*P*)-**3**-C_16_ with the C_16_ terminal groups; Section 6.2 describes a combination of (*P*)–tetramer (*P*)-**3** and (*M*)–hexamer (*M*)-**5**.

### 6.1. Unstable Concentration Oscillations Involving Equilibrium Touching

#### 6.1.1. Transformation from Unstable Concentration Oscillations to Stable Ones

**Figure 17:**
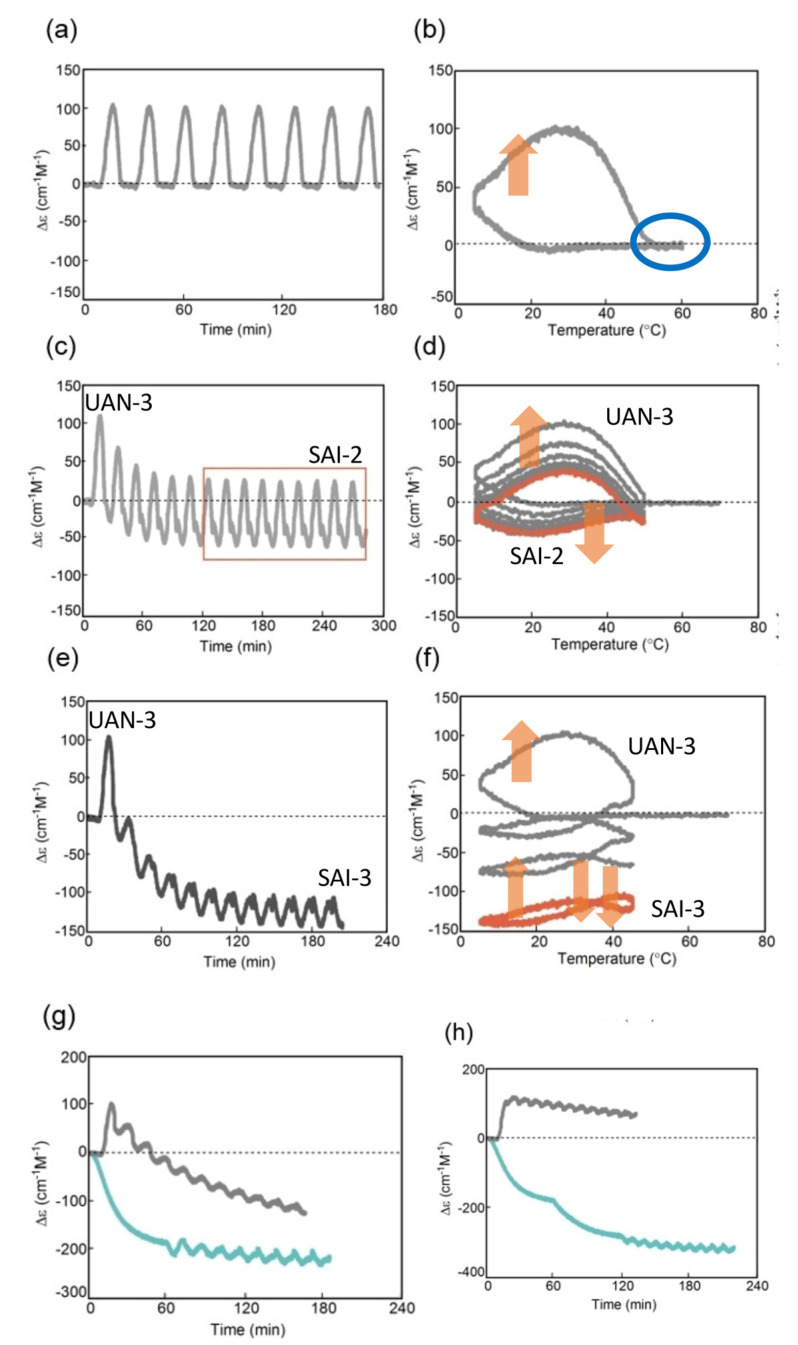
Concentration oscillations of (*P*)–**3**-C_16_/(*M*)–**4** in fluorobenzene (0.5 mM) by different ranges of temperature oscillations at a rate of 5.0 K min^−1^. (**a**,**b**) A 60/5 °C temperature oscillation providing SAO-2, (**c**,**d**) a 50/5 °C temperature oscillation providing a transformation from UAN-3 to SAI-2, (**e**,**f**) a 45/5 °C temperature oscillation providing a transformation from UAN-3 to SAI-3, (**g**) a 40/5 °C temperature oscillation, and (**h**) a 30/5 °C temperature oscillation. Red square in (**c**) and red line in (**d**) indicate SAI-2; first three cycles of UAN-3 (gray lines) and SAI-3 (red lines) are shown in (**f**); orange bold arrows indicate self-catalytic reactions; blue circle indicates equilibrium overlapping; blue lines in (**g**,**h**) indicate reaction shortcut phenomena. Reproduced from ref. [20] with permission from Wiley.

A mixture of (*P*)-**3**-C_16_ and (*M*)-**4** in fluorobenzene (0.5 mM) forms **B**_2_(A) and **C**_2_(A) with the enantiomeric helical senses with the negative and positive Δε at 314 nm, respectively, in which **B**_2_(A) is thermodynamically more stable than **C**_2_(A) [20]. A mixture containing fully dissociated 2**A**_2_(A) at 70 °C with Δε 0 cm^−1^ M^−1^ was cooled to 5 °C at a rate of 5.0 K min^−1^, and a 60/5 °C temperature oscillation was provided (Figure 17a,b). SAO-2 appears between Δε 0 and +100 cm^−1^ M^−1^, which indicates the interconversion between **C**_2_(A) and 2**A**_2_(A). Δε increases during heating in a cycle between 15 and 30 °C but not during cooling, which shows a significant delay of 2**A**_2_(A) + **C**_2_(A)→2**C**_2_(A) versus temperature change. Equilibrium overlapping at above 50 °C provides a stable concentration oscillation SAO-2 (Figure 17b, blue circle).

A 50/5 °C temperature oscillation provides UAN-3 with a gradient retarded concentration oscillation not involving equilibrium overlapping, which is transformed into SAI-2 between Δε +21 and −60 cm^−1^ M^−1^ (Figure 17c,d). A transformation from UAN-3 with the positive Δε to SAI-2 with the positive/negative Δε occurs owing to the competition of two self-catalytic reactions, in which 2**A**_2_(A) + **B**_2_(A)→2**B**_2_(A) occurs at 35 °C during cooling in a cycle and 2**A**_2_(A) + **C**_2_(A)→2**C**_2_(A) occurs at 20 °C during heating (Figure 17d, orange bold arrows).

A 45/5 °C temperature oscillation initially provides UAN-3 owing to 2**A**_2_(A) + **C**_2_(A)→2**C**_2_(A) during heating in a cycle. Repeating cycles increases the strength of 2**A**_2_(A) + **B**_2_(A)→2**B**_2_(A) (Figure 17e,f), and a doubled-frequency concentration oscillation SAI-3 with figure-eight hysteresis occurs between Δε −120 and −150 cm^−1^ M^−1^ after eight cycles. SAI-3 involves the competition of 2**A**_2_(A) + **B**_2_(A)→2**B**_2_(A) and 2**A**_2_(A) + **C**_2_(A)→2**C**_2_(A) in a cycle between 30 and 40 °C.

A 40/5 °C temperature oscillation provides UAN-2, which exhibits gradual decrease in Δε by repeated cycles (Figure 17g, grey line). The decrease occurs owing to the initial formation of **C**_2_ (A) with the positive Δε and subsequent slow formation of thermodynamically stable **B**_2_(A) with the negative Δε. A 30/5 °C temperature oscillation provides another UAN-2 with a slower decrease in Δε by repeated cycles (Figure 17h, grey line). Stable concentration oscillations are not reached after 10 cycles of the 40/5 °C and 30/5 °C temperature oscillations.

The above experiments show that the unstable and stable concentration oscillations are significantly affected by temperature ranges with a fixed low temperature of 5 °C and variable high temperatures in a cycle (Figure 17). A general tendency to switch from UAN-3 involving 2**A**_2_(A) + **C**_2_(A)→2**C**_2_(A) to SAI-3 involving 2**A**_2_(A) + **C**_2_(A)→2**C**_2_(A) and 2**A**_2_(A) + **B**_2_(A)→2**B**_2_(A) appears. A resonance phenomenon also appears, in which SAI-3 with the doubled-frequency concentration oscillations occurs only by a 45/5 °C temperature oscillation but not by 50/5 and 40/5 °C.

**Figure 18:**
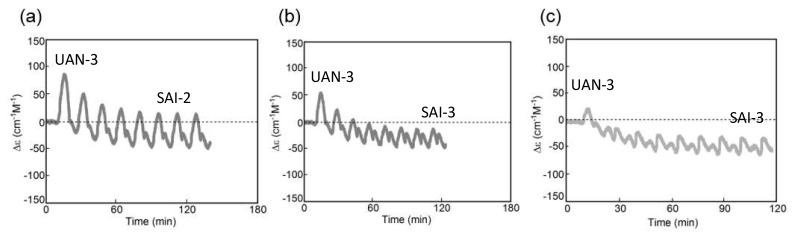
Concentration oscillations of (*P*)–**3**–C_16_/(*M*)-**4** in fluorobenzene (0.5 mM) at a rate of 5.0 K min^−1^ starting from 70 °C: (**a**) A 50/10 °C temperature oscillation providing a transformation from UAN-3 to SAI-2; (**b**) 50/15 °C and (**c**) 50/20 °C temperature oscillations providing transformations from UAN-3 to SAI-3. Reproduced from ref. [20] with permission from Wiley.

Another series of 50/5 °C, 50/10 °C, 50/15 °C, and 50/20 °C temperature oscillations is provided. The 50/5 °C and 50/10 °C temperature oscillations provide the transformations from UAN-3 to SAI-2 (Figure 17c and Figure 18a), and 50/15 °C and 50/20 °C temperature oscillations provide the transformations from UAN-3 to SAI-3 (Figure 18b,c). Then, the doubled–frequency concentration oscillations are not significantly affected by the temperature oscillations in temperature ranges of a fixed high temperature of 50 °C and variable low temperatures in a cycle. The results of these temperature oscillations indicate that the competition of 2**A**_2_(A) + **C**_2_(A)→2**C**_2_(A) and 2**A**_2_(A) + **B**_2_(A)→2**B**_2_(A) occurs between 30 and 40 °C.

#### 6.1.2. Convergence of Different Unstable Concentration Oscillations to a Single Stable Concentration Oscillation

**Figure 19:**
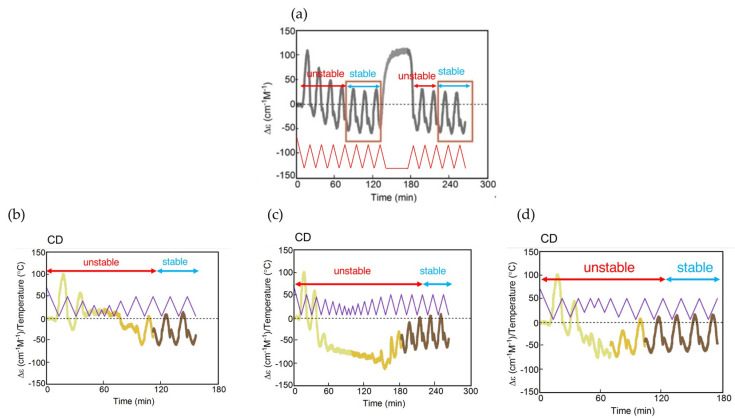
Stable nature of SAI-2 of (*P*)-**3**-C_16_/(*M*)-**4** in fluorobenzene (0.5 mM) by a 50/5 °C temperature oscillation at a rate of 5.0 K min^−1^ starting from 70 °C. (**a**) A 50/5 °C temperature oscillation is provided followed by settling at 5 °C for 45 min, and then 50/5 °C temperature oscillation is provided. Red squares indicate SAI-2. (**b**) A pseudo-periodic temperature change is provided changing the range from 50/5 to 20/5 and then to 50/5 °C with a fixed low temperature of 5 °C, which is followed by a 50/5 °C temperature oscillation. (**c**) Another pseudo-periodic temperature change is provided changing the range from 50/5 to 20/5 and then to 50/5 °C with a fixed low temperature of 5 °C, which is followed by a 50/5 °C temperature oscillation. (**d**) A pseudo-periodic temperature change is provided changing the range from 50/5 to 50/30 and then to 50/5 °C with a fixed high temperature of 50 °C, which is followed by a 50/5 °C temperature oscillation. Red lines in (**a**) and purple lines in (**b**–**d**) indicate temperature oscillations. Reproduced from ref. [20] with permission from Wiley.

Stable nature of a stable concentration oscillation against thermal perturbation and thermal history is described below, which shows the convergence of different unstable concentration oscillations to a single stable concentration oscillation. When a 50/5 °C temperature oscillation was provided to (*P*)-**3**-C_16_/(*M*)-**4** in fluorobenzene (0.5 mM) at a rate of 5.0 K min^−1^, SAI-2 between −50 and +30 cm^−1^ M^−1^ occurred after four cycles (Figure 19a). The mixture was cooled to 5 °C and settled for 45 min, which exhibited Δε approximately +100 cm^−1^ M^−1^. Then, another 50/5 °C temperature oscillation was provided, which regenerated the original SAI-2 after three cycles (Figure 19a, red square).

A pseudo-periodic temperature oscillation is provided, which involves a gradual change of the temperature range from 50/5 to 20/5 °C and then to 50/5 °C with a fixed low temperature of 5 °C and variable high temperatures. A complex unstable concentration oscillation occurs at Δε approximately 0 cm^−1^ M^−1^ with a small amplitude (Figure 19b). When a 50/5 °C temperature oscillation is provided, the same SAI-2 shown in Figure 19a appears after two cycles. Another slightly different pseudo-periodic temperature change also provides the same SAI-2 (Figure 19c). A different pseudo-periodic temperature change is provided, which involves a gradual change of temperature range from 50/5 to 50/30 °C and then to 50/5 °C with a fixed high temperature of 50 °C. The subsequent 50/5 °C temperature oscillation again provides the same SAI-2 (Figure 19d). The results show that different unstable concentration oscillations converge to the same SAI-2 under a 50/5 °C temperature oscillation, and the occurrence of the SAI-2 is not affected by their thermal histories.

**Figure 20:**
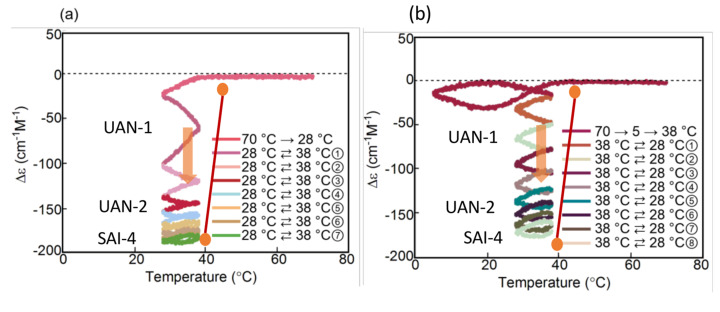
Concentration oscillations of (*P*)–**3**–C_16_/(*M*)–**4** in fluorobenzene (0.5 mM) by a 38/28 °C temperature oscillation at a rate of 2.0 K min^−1^ providing a transformation from UAN-1 to UAN-2 and then to SAI-4. (**a**) Starting from 70 °C and (**b**) starting from 70 °C and cooling to 5 °C. Orange lines indicate equilibrium curve between 40 and 45 °C; orange bold arrows indicate a self-catalytic reaction 2**A**_2_(A) + **B**_2_(A)→2**B**_2_(A). Reproduced from ref. [45].

Periodic temperature oscillations over a narrow range induces a transformation from UAN-1 to UAN-2 and then to SAI-4 (Figure 6a and Figure 20), which also provide very similar SAI-4 irrespective of their thermal histories. When 2**A**_2_(A) at 70 °C is cooled to 28 °C, and a 38/28 °C temperature oscillation is provided at a rate of 2.0 K min^−1^, UAN-1 is transformed to UAN-2 and to SAI-4 with Δε approximately −180 cm^−1^ M^−1^ close to the equilibrium curve (Figure 20a) [43]. The transformation occurs through the decrease in the strength of 2**A**_2_(A) + **B**_2_(A)→2**B**_2_(A) from a strong one in UAN-1 between 30 and 40 °C to a minimal one in SAI-4. A different thermal history of temperature oscillation is provided by cooling 2**A**_2_(A) from 70 °C to 5 °C followed by the 38/28 °C temperature oscillation. The transformation from UAN-1 to UAN-2 and then to SAI-4 occurs and provides SAI-4 with Δε approximately −170 cm^−1^ M^−1^ close to the equilibrium curve (Figure 20b). Of note is that two procedures with different thermal histories provide very similar SAI-4 under a 38/28 °C temperature oscillation.

#### 6.1.3. Equilibrium Touching

**Figure 21:**
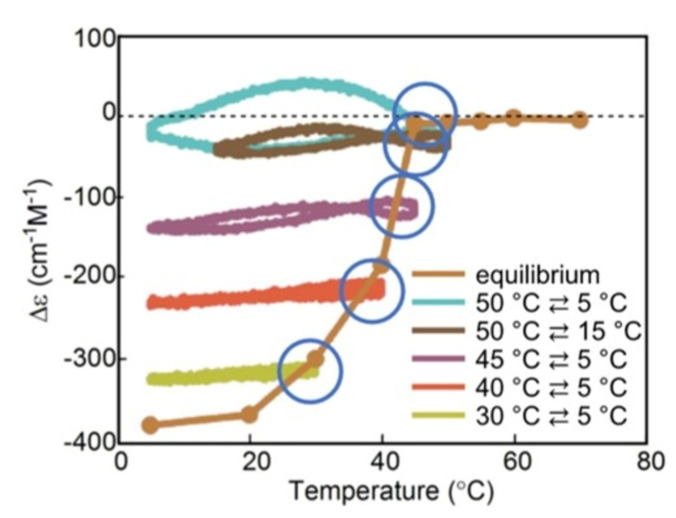
Equilibrium touching by stable concentration oscillations of (*P*)–**3**–C_16_/(*M*)–**4** in fluorobenzene (0.5 mM) at a rate of 5.0 K min^−1^, which are taken from Figure 17 and Figure 18b. Blue circles indicate equilibrium touching. Orange line indicates equilibrium curve. Reproduced from ref. [20] with permission from Wiley.

The above 50/5, 50/15, 45/5, 40/5, and 30/5 °C temperature oscillations provide different stable concentration oscillations through unstable concentration oscillations (Figure 17 and Figure 18b): A 50/5 °C temperature oscillation provides SAI-2; 50/15 and 45/5 °C temperature oscillations provide SAI-3; 40/5 and 30/5 °C temperature oscillations provide SAI-4 (Figure 21). A larger hysteresis area appeared by the 50/5 °C temperature oscillation owing to the competition of 2**A**_2_(A) + **B**_2_(A)→2**B**_2_(A) and 2**A**_2_(A) + **C**_2_(A)→2**C**_2_(A), and a smaller area by the 30/5 °C temperature oscillation owing to the minimal self-catalytic reactions. When these stable concentration oscillations are compared with the equilibrium curve, it is noted that they intersect or make contact with the equilibrium curve at high temperatures (Figure 21, blue circles). This phenomenon exhibited by a set of stable concentration oscillations was previously named equilibrium touching [20]. The results indicate that unstable concentration oscillations occur in the direction towards the equilibrium curve (Figure 17 and Figure 18b) and provide stable concentration oscillations involving equilibrium touching. The phenomenon of equilibrium touching shows an effect to stabilize unstable concentration oscillations, which transforms unstable concentration oscillations to stable ones.

Transformation from unstable to stable concentration oscillations can significantly be accelerated by an initial cooling procedure, and the acceleration phenomenon is called reaction shortcut [57]. The transformation without the cooling procedure is very slow, as shown by grey lines in Figure 17g,h. However, when 2**A**_2_(A) at 70 °C is initially cooled to 40 °C during 60 min and provided with a 40/5 °C temperature oscillation, a stable concentration oscillation at approximately Δε −200 cm^−1^ M^−1^ occurs after eight cycles (Figure 17g, blue line). The cooling procedure eliminates the initial formation of **C**_2_(A) exhibiting the positive Δε, and significantly accelerates the formation of **B**_2_(A) with the negative Δε. A 30/5 °C temperature oscillation also shows a similar trend (Figure 17h, grey and blue lines). The procedures are employed to obtain stable concentration oscillations by 40/5 °C and 30/5 °C temperature oscillations (Figure 21).

#### 6.1.4. Domains of Self-Catalytic Reactions of (P)-3-C_16_/(M)-4

**Figure 22:**
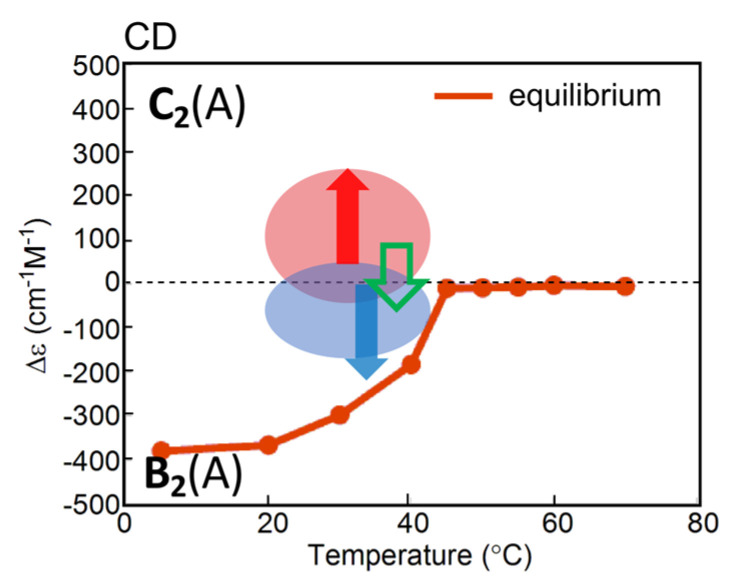
Domains of self-catalytic reactions of (*P*)–**3**–C_16_/(*M*)–**4** in fluorobenzene (0.5 mM). Red circle indicates 2**A**_2_(A) + **B**_2_(A)→2**B**_2_(A) domain, and red bold arrow indicates its direction; blue circle indicates 2**A**_2_(A) + **C**_2_(A)→2**C**_2_(A) domain, and blue bold arrow indicates its direction; green arrow indicates the switching of the domains from 2**A**_2_(A) + **C**_2_(A)→2**C**_2_(A) to 2**A**_2_(A) + **B**_2_(A)→2**B**_2_(A): red line indicates equilibrium curve.

Domains of 2**A**_2_(A) + **B**_2_(A)→2**B**_2_(A) and 2**A**_2_(A) + **C**_2_(A)→2**C**_2_(A) are shown by the Δε/temperature profile of (*P*)-**3**-C_16_/(*M*)-**4**, which are compared with the equilibrium curve (Figure 7 and Figure 22). A strong 2**A**_2_(A) + **B**_2_(A)→2**B**_2_(A) occurs at Δε between −50 and −150 cm^−1^ M^−1^ (Figure 22, blue circle), which exhibits UAN-1 (Figure 20); 2**A**_2_(A) + **C**_2_(A)→2**C**_2_(A) occurs at Δε close to +50 cm^−1^ M^−1^ (Figure 22, red circle), which exhibits SAO-2 (Figure 17a,b). Both reactions occur between 20 and 40 °C. Of note is that 2**A**_2_(A) + **C**_2_(A)→2**C**_2_(A) proceeds in the direction away from the equilibrium. SAI-3 with doubled-frequency concentration oscillations occurs at Δε close to 0 cm^−1^ M^−1^ (Figure 17e and Figure 18b,c) owing to the competition of 2**A**_2_(A) + **B**_2_(A)→2**B**_2_(A) and 2**A**_2_(A) + **C**_2_(A)→2**C**_2_(A), which is shown by the common domain of blue and red circles (Figure 22). The transformations in Figure 17 shows the switching from 2**A**_2_(A) + **C**_2_(A)→2**C**_2_(A) to 2**A**_2_(A) + **B**_2_(A)→2**B**_2_(A) by repeated cycles, which involves the change of the domains from a red to blue one as it approaches the equilibrium curve (Figure 7b and Figure 22, green arrow).

### 6.2. Unstable Concentration Oscillations Involving Equilibrium Sliding

In Section 6.2, unstable concentration oscillations of a mixture of aminomethylene (*P*)–tetramer (*P*)–**3** and (*M*)–hexamer (*M*)–**5** are described, which show different features from those of (*P*)-**3**-C_16_/(*M*)-**4** described in Section 6.1. Unstable concentration oscillations of (*P*)–**3**/(*M*)–**5** involve a reaction system of 2**A**_3_(A)
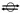
**B**_3_(E) and 2**A**_3_(A)
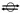
**C**_3_(A) with competitive 2**A**_3_(A) + **B**_3_(A)→2**B**_3_(A) and 2**A**_3_(A) + **C**_3_(A)→2**C**_3_(A), which form hetero-double-helices **B**_3_(A) and **C**_3_(A) with the enantiomeric senses exhibiting the negative and positive Δε at 315 nm, respectively [58]. Note that **C**_3_(A) is thermodynamically more stable than **B**_3_(A), which is in contrast to (*P*)-**3**-C_16_/(*M*)-**4**, in which **B**_2_(A) is thermodynamically more stable than **C**_2_(A). Equilibrium sliding occurs by (*P*)–**3**/(*M*)–**5**, and cooling and heating curves in unstable concentration oscillations repeatedly intersect the equilibrium curves owing to 2**A**_3_(A) + **C**_3_(A)→2**C**_3_(A) along the equilibrium curves (Figure 5). Additionally, a stable concentration oscillation occurs without contact with the equilibrium curve, which is transformed to an unstable concentration oscillation and eventually to another stable concentration oscillation.

#### 6.2.1. Unstable Concentration Oscillations under Different Temperature Change Rates

**Figure 23:**
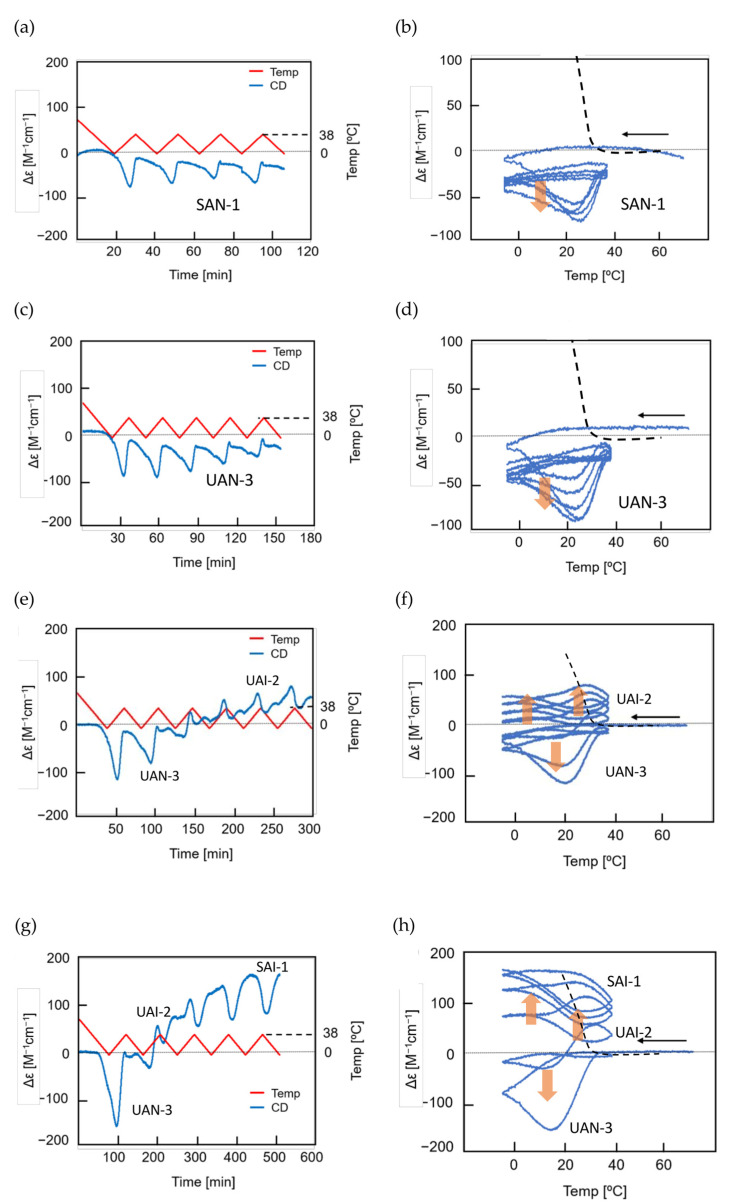
Unstable concentration oscillations of (*P*)-**3**/(*M*)-**5** in toluene (0.25 mM) induced by 38/−5 °C temperature oscillations at the rates of (**a**,**b**) 4.0 K min^−1^ providing SAN-1, (**c**,**d**) 3.0 K min^−1^ providing UAN-3, (**e**,**f**) 2.0 K min^−1^ providing a transformation from UAN-3 to UAI-2, and (**g**,**h**) 1.0 K min^−1^ providing a transformation from UAN-3 to UAI-2 and then to SAI-1. Orange bold arrows indicate self-catalytic reactions; dashed lines indicate equilibrium curves. Reproduced from ref. [58].

A mixture of (*P*)–**3** and (*M*)–**5** in toluene (0.25 mM) with fully dissociated 2**A**_3_(A) at 70 °C was cooled to −5 °C at a rate of 4.0 K min^−1^, and a 38/−5 °C temperature oscillation was provided. SAN-1 involving retarded concentration oscillation occurs after two cycles, which exhibits a decrease in Δε at 10 °C in a cycle during heating owing to 2**A**_3_(A) + **B**_3_(A)→2**B**_3_(A) (Figure 23a,b). SAN-1 occurs without contact with the equilibrium curve, which is a phenomenon of equilibrium noncontact. At a rate of 3.0 K min^−1^, UAN-3 involving gradient retarded concentration oscillation with swing hysteresis occurs, in which Δε increases at 30 °C in a cycle owing to 2**A**_3_(A) + **C**_3_(A)→2**C**_3_(A) in the direction to form thermodynamically stable **C**_3_(A) (Figure 23c,d). The concentration oscillation phenomena suggest that a stable concentration SAN-1 at a rate of 4.0 K min^−1^ can be transformed into an unstable concentration oscillation UAN-3 by changing the rate to 3.0 K min^−1^. The transformation phenomenon is in contrast to other transformations, which occur from an unstable concentration to a stable one.

Decrease in the rate to 2.0 K min^−1^ exhibits a transformation from UAN-3 to UAI-2 (Figure 23e,f). After the initial three cycles of UAN-3 with an increase in Δε at 30 and 5 °C in a cycle, UAI-2 involving equilibrium sliding occurs (Figure 5e). UAI-2 owing to 2**A**_3_(A) + **C**_3_(A)→2**C**_3_(A) occurs upward by repeated cycles along the equilibrium curve, as shown in the Δε/temperature profile, which does not provide a stable concentration oscillation after seven cycles.

At a rate of 1.0 K min^−1^, UAN-3 is transformed to UAI-2 and then to SAI-1 (Figure 23g,h). The first cycle of UAN-3 shows decrease in Δε at 15 °C in a cycle during heating owing to 2**A**_3_(A) + **C**_3_(A)→2**C**_3_(A). Then, UAN-3 is transformed into UAI-2 with two Δε maxima at 30 °C in a cycle during heating and at 5 °C during cooling, which exhibits equilibrium sliding. UAI-2 involving doubled-frequency concentration oscillation occurs owing to the competition of 2**A**_3_(A) + **B**_3_(A)→2**B**_3_(A) and 2**A**_3_(A) + **C**_3_(A)→2**C**_3_(A). Eventually, SAI-1 with 2**A**_3_(A) + **B**_3_(A)→2**B**_3_(A) involving equilibrium intersecting occurs under a balanced state. The transformation involves switching from 2**A**_3_(A) + **C**_3_(A)→2**C**_3_(A) to 2**A**_3_(A) + **B**_3_(A)→2**B**_3_(A).

Thus, a significantly different nature of stable concentration oscillations occurs by the 38/−5 °C temperature oscillations under different temperature change rates, as indicated by SAN-1 at a rate of 4.0 K min^−1^ with the negative Δε (Figure 23a,b) and SAI-1 at 1.0 K min^−1^ with the positive Δε (Figure 23g,h). It is because SAI-1 occurs at a slower rate of 1.0 K min^−1^ close to the equilibrium curve, and the slow rate provides a sufficient period of time to approach equilibrium.

A resonance phenomenon appears with regard to the frequency, in which the doubled-frequency concentration oscillation UAI-2 involving two Δε maxima in a cycle occurs at a rate of 2.0 K min^−1^ but not at 1.0, 3.0, and 4.0 K min^−1^ (Figure 23e). It is because of the competitive 2**A**_3_(A) + **B**_3_(A)→2**B**_3_(A) and 2**A**_3_(A) + **C**_3_(A)→2**C**_3_(A) at 2.0 K min^−1^. In contrast, 2**A**_3_(A) + **B**_3_(A)→2**B**_3_(A) predominates at 1.0 K min^−1^, and 2**A**_3_(A) + **C**_3_(A)→2**C**_3_(A) at 3.0 and 4.0 K min^−1^.

Another interesting phenomenon is that the decrease in the temperature change rate from 4.0 to 1.0 K min^−1^ induces the transformation from a stable concentration oscillation SAN-1 involving equilibrium noncontact to an unstable concentration oscillation UAI-2 involving equilibrium sliding, which eventually is transformed to a stable concentration oscillation SAI-1 involving equilibrium intersecting. The transformation between two stable concentration oscillations can occur under the nonequilibrium states.

The transformations from unstable to stable concentration oscillations exhibit notable nonequilibrium phenomena including equilibrium sliding, resonance phenomenon, transformation from a stable concentration oscillation to another one, and their high sensitivity to environmental conditions.

#### 6.2.2. Unstable Concentration Oscillations under Different Temperature Ranges

**Figure 24:**
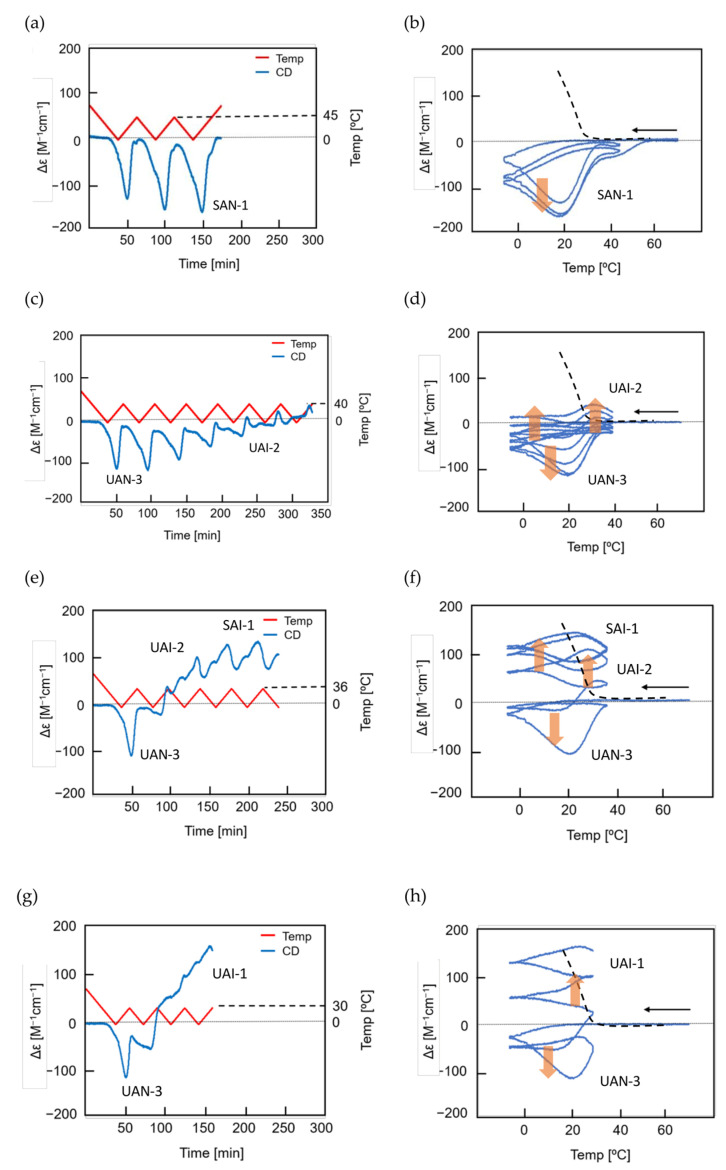
Transformations of concentration oscillations of (*P*)-**3**/(*M*)-**5** in toluene (total 0.25 mM) at a rate of 2.0 K min^−1^ by (**a**,**b**) a 45/−5 °C temperature oscillation providing SAN-1, (**c**,**d**) a 40/−5 °C providing a transformation from UAN-3 to UAI-2, (**e**,**f**) a 36/−5 °C providing a transformation from UAN-3 to UAI-2 to SAI-1, and (**g**,**h**) a 30/−5 °C providing a transformation from UAN-3 to UAI-1. Orange bold arrows indicate self-catalytic reactions; dashed lines indicate equilibrium curves. Reproduced from ref. [58].

Section 6.2.1 described concentration oscillations provided by temperature oscillations at different temperature change rates. When temperature oscillations are provided at different temperature ranges, a similar trend of transformations from unstable concentration oscillations to stable ones occurs. However, a notable phenomenon can also occur, as will be described in Section 6.2.2. A 45/−5 °C temperature oscillation at a rate of 2.0 K min^−1^ provides SAN-1 involving 2**A**_3_(A) + **B**_3_(A)→2**B**_3_(A) (Figure 24a,b). A 40/−5 °C temperature oscillation provides a transformation from UAN-3 to UAI-2 involving equilibrium sliding owing to 2**A**_3_(A) + **C**_3_(A)→2**C**_3_(A) (Figure 24c,d). A 36/−5 °C temperature oscillation provides a transformation from UAN-3 to UAI-2 and then to SAI-2 (Figure 24e,f). The transformations at different temperature ranges in general shows a similar tendency with those of temperature change rates (Figure 23). A notable observation is that a 30/−5 °C temperature oscillation provides UAI-1 involving zigzag hysteresis and equilibrium sliding along the equilibrium curve (Figure 24g,h). It is because of a strong 2**A**_3_(A) + **C**_3_(A)→2**C**_3_(A) at close to 30 °C.

#### 6.2.3. Domains of self-catalytic reactions of (P)-3/(M)-5

**Figure 25:**
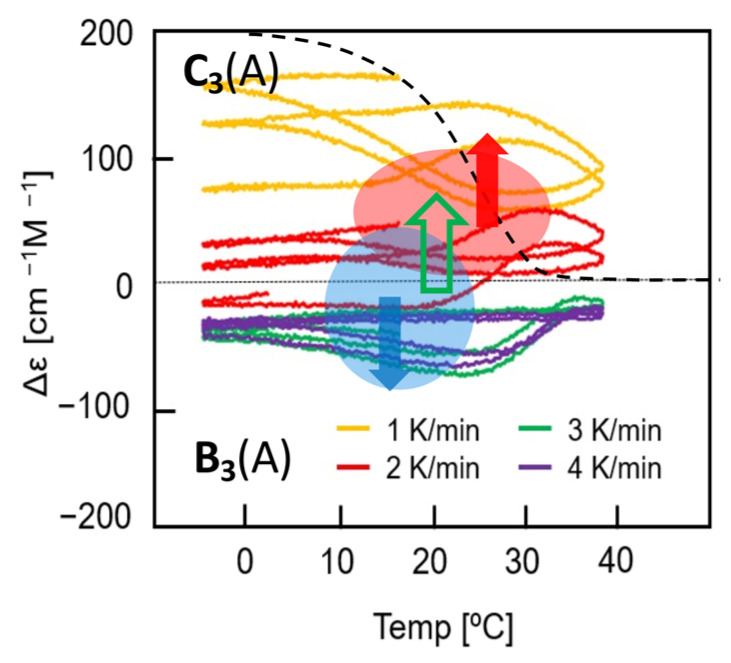
Domains of self-catalytic reactions of (*P*)-**3**/(*M*)-**5** in toluene (total 0.25 mM) by a 40/−5 °C temperature oscillation. A part of Figure 21 is shown. Red circle indicates a strong 2**A**_3_(A) + **C**_3_(A)→2**C**_3_(A) domain, and red bold arrow indicates its direction; blue circle indicates a 2**A**_3_(A) + **B**_3_(A)→2**B**_3_(A) domain, and blue bold arrow indicates its direction; dashed line indicates equilibrium curve; green arrow indicates the switching of the domains of 2**A**_3_(A) + **B**_3_(A)→2**B**_3_(A) to 2**A**_3_(A) + **C**_3_(A)→2**C**_3_(A).

Domains of self-catalytic reactions 2**A**_3_(A) + **B**_3_(A)→2**B**_3_(A) and 2**A**_3_(A) + **C**_3_(A)→2**C**_3_(A) of (*P*)-**3**/(*M*)-**5** are shown in Δε/temperature profiles. A strong 2**A**_3_(A) + **C**_3_(A)→2**C**_3_(A) at 30 °C with approximately Δε +100 cm^−1^ M^−1^ exhibits UAI-1 involving equilibrium sliding (Figure 24h and Figure 25, red circle). Additionally, 2**A**_3_(A) + **B**_3_(A)→2**B**_3_(A) occurs at close to 10 °C with Δε approximately −50 cm^−1^ M^−1^, which provides SAN-1 (Figure 24b and Figure 25, blue circle). Competition of 2**A**_3_(A) + **B**_3_(A)→2**B**_3_(A) and 2**A**_3_(A) + **C**_3_(A)→2**C**_3_(A) occurs at Δε approximately +50 cm^−1^ M^−1^ at the common domain of red and blue circles, which exhibits UAI-2 (Figure 24f and Figure 25). The self-catalytic reactions are highly sensitive to temperatures, as shown by the domains. It is noted that the transformations in Figure 23 and Figure 24 show a general tendency of switching from 2**A**_3_(A) + **B**_3_(A)→2**B**_3_(A) to 2**A**_3_(A) + **C**_3_(A)→2**C**_3_(A) by repeated cycles. It is because the transformations involve the change of the domains from blue to red ones as they approach the equilibrium curve (Figure 7c and Figure 25, green arrow).

#### 6.2.4. Mechanism of Doubled-Frequency Concentration Oscillations

**Figure 26:**
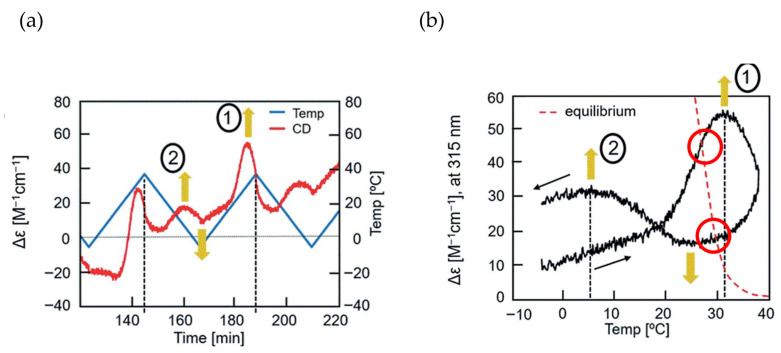
UAI-2 of (*P*)-**3**/(*M*)-**5** in toluene (total 0.25 mM) at a rate of 2.0 K min^−1^ by a 38/−5 °C temperature oscillation shown in (**a**) Δε/time profiles and (**b**) Δε/temperature profiles. Two Δε maxima 1 and 2 and a minimum are shown. Dashed red lines in (**b**) indicate equilibrium curves; orange arrows indicate self-catalytic reactions; red circles indicate equilibrium intersections. Reproduced from ref. [58].

A mechanistic explanation of an unstable doubled-frequency concentration oscillation UAI-2 exhibiting two maxima of Δε in a cycle is described below, which occurs owing to the competition of 2**A**_3_(A) + **B**_3_(A)→2**B**_3_(A) and 2**A**_3_(A) + **C**_3_(A)→2**C**_3_(A) (Figure 26) [58]. A 38/−5 °C temperature oscillation provides a cycle with two maxima 1 and 2 of Δε at approximately 30 and 5 °C during heating and a minimum at 20 °C during cooling, which occurs at Δε close to 0 cm^−1^ M^−1^. Because Δε is proportional to [**C**_3_(A)] − [**B**_3_(A)], the maxima appear by the increase in **C**_3_(A) owing to 2**A**_3_(A) + **C**_3_(A)→2**C**_3_(A) and the minimum by the increase in **B**_3_(A) owing to 2**A**_3_(A) + **B**_3_(A)→2**B**_3_(A) (Figure 26, orange arrows). A difference in the temperature dependences of 2**A**_3_(A) + **B**_3_(A)→2**B**_3_(A) and 2**A**_3_(A) + **C**_3_(A)→2**C**_3_(A) plays an important role in the doubled-frequency phenomenon. Equilibrium sliding occurs by intersecting the equilibrium curve twice during a cycle (Figure 26b, red circles). Related discussions on the mechanism of stable doubled-frequency concentration oscillations SAI-3 with figure-eight hysteresis have been described by concentrations of 2**A**_3_(A), **B**_3_(A), and **C**_3_(A), which are determined using Δε obtained by CD and ε obtained by UV-vis [43].

#### 6.2.5. Comparison of Unstable Concentration Oscillations of (P)-3-C_16_/(M)-4 and (P)-3/(M)-5

Section 6.1 described (*P*)-**3**-C_16_/(*M*)-**4**, which is a combination of aminomethylene (*P*)-tetramer and (*M*)-pentamer, and Section 6.2 described (*P*)-**3**/(*M*)-**5**, which is a combination of (*P*)-tetramer and (*M*)-hexamer (Figure 8). In addition to the different numbers of helicene units, (*P*)-**3**-C_16_/(*M*)-**4** possesses C_16_ alkyl group at the terminal position whereas (*P*)-**3**/(*M*)-**5** does not. Although both combinations form hetero-double-helices **B** and **C** with the enantiomeric helical senses, their behavior in concentration oscillations differ. (1) The relative thermodynamic stability of **B** and **C** inverts: **B**_2_(A) is stable in (*P*)-**3**-C_16_/(*M*)-**4** and **C**_3_(A) is stable in (*P*)-**3**/(*M*)-**5** (Figure 22 and Figure 25), and the long alkyl groups affect the thermodynamic stability of the helical sense [43]. (2) Equilibrium sliding occurs in (*P*)-**3**/(*M*)-**5** owing to 2**A**_3_(A) + **C**_3_(A)→2**C**_3_(A) close to the equilibrium curve (Figure 25), whereas (*P*)-**3**-C_16_/(*M*)-**4** exhibits equilibrium touching owing to 2**A**_3_(A) + **B**_2_(A)→2**B**_2_(A) away from the equilibrium curve (Figure 22). (3) Property of metastable states differs: the metastable **B**_3_(A) in (*P*)-**3**/(*M*)-**5** is relatively stable and exhibits stable concentration oscillation SAN-1 involving equilibrium noncontact (Figure 24a,b), whereas metastable **C**_2_(A) in (*P*)-**3**-C_16_/(*M*)-**4** is relatively unstable and does not exhibit stable concentration oscillation (Figure 17 e,f). This is an advantage of the helicene oligomers, properties of which can be fine-tuned by the modification of their molecular structures.

## 7. Concentration Oscillations in Aqueous Solutions

Concentration oscillations induced by temperature oscillations can be used to analyze the nature of reactions, and an example is described below. A reaction of molecules dispersed in aqueous solution is affected by the interactions between substrate/product molecules and water molecules and interactions between water molecules. Ethynyl (*M*)-tetramer (*M*)-**6**-TEG and (*P*)-pentamer (*P*)-**7**-TEG possessing the tetraethylene glycolyl (TEG) groups at the terminal positions show high solubility in the mixtures of water and THF and form hetero-double-helix **B**(E) with the negative Δε at 369 nm and random-coil 2**A**(E) with Δε 0 cm^−1^ M^−1^ [59]. The 2**A**(E)
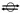
**B**(E) in water/THF is switched between stable and unstable concentration oscillations, which are induced by a small change in water content.

**Figure 27:**
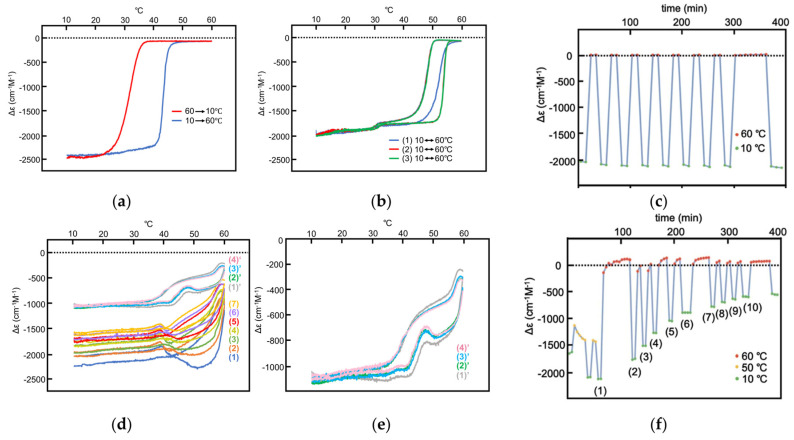
Stable concentration oscillations of (*M*)-**6**-TEG/(*P*)-**7**-TEG in water/THF (0.010 mM) by a 60/10 °C temperature oscillation at a rate of 1.0 K min^−1^ in (**a**) 25% and (**b**) 30% water/THF. (**c**) A concentration oscillation by a square temperature oscillation between 60 and 10 °C in 30% water/THF. (**d**) An unstable concentration oscillation by a 60/10 °C temperature oscillation in 33% water/THF and (**e**) an expansion of four cycles of a stable concentration oscillation. (**f**) An unstable concentration oscillation provided by a square temperature oscillation between 60 and 10 °C in 33% water/THF. Numbers of heating and cooling cycles are shown in parentheses. Reproduced from ref. [59].

A mixture of (*M*)-**6**-TEG and (*P*)-**7**-TEG in different ratios of water and THF (0.01 mM) is provided with a 60/10 °C temperature oscillation at a rate of 1.0 K min^−1^. In 25% water/THF, a solution containing fully dissociated 2**A**(E) with Δε 0 cm^−1^ M^−1^ is formed at high temperatures, as indicated by the flat curve of high-temperature limit states, which involves equilibrium overlapping (Figure 27a). A 60/10 °C temperature oscillation at a rate of 1.0 K min^−1^ provides a stable concentration oscillation between Δε 0 and −2000 cm^−1^ M^−1^. Upon cooling and heating, a significant decrease and increase in Δε within less than 10 °C of the temperature change occur between high- and low-temperature limit states, which suggests the involvement of 2**A**(E) + **B**(E)→2**B**(E). In 30% water/THF, another stable concentration oscillation occurs involving equilibrium overlapping at high temperatures (Figure 27b). The cooling and heating curves are shifted to the high temperatures compared to those in 25% water/THF. The stable nature of the concentration oscillations is also determined by providing a square temperature oscillation between 60 and 10 °C (Figure 27c).

It is confirmed that 2**A**(E)
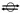
**B**(E) does not occur in THF, which shows a critical role of water in the formation of **B**(E) inducing strong hydrophobic interactions. The formation of **B**(E) at a low concentration of 0.01 mM in water/THF is consistent with the presence of the interpretations, which is in contrast to high concentrations above 0.2 mM required in organic solvents, as described in Section 4, Section 5 and Section 6. This is a molecular event dispersed in solution as determined by DLS, and aggregation of **B**(E) is minimal.

A small increase in the water content from 30 to 33% provides an unstable concentration oscillation. A solution in 33% water/THF at 10 °C, which contains **B**(E) with Δε approximately −2200 cm^−1^ M^−1^, is provided with a 60/10 °C temperature oscillation at a rate of 1.0 K min^−1^. Heating to 60 °C provides Δε approximately −1000 cm^−1^ M^−1^ and cooling to 10 °C Δε approximately −2000 cm^−1^ M^−1^ (Figure 27d,e). An unstable concentration oscillation occurs with a gradual increase in Δε by repeated cycles. In every cycle, no reaction occurs below 35 °C, as shown by the coincidence of the flat cooling and heating curves, and hysteresis appears above 35 °C. A stable concentration oscillation occurs after 17 cycles, in which the balanced state between reactions and temperature change is achieved. A transformation from an unstable to a stable concentration oscillation is determined by providing a square temperature oscillation between 60 and 10 °C (Figure 27f).

In 25 and 30% water/THF, stable concentration oscillations occur involving equilibrium overlapping; in 33% water/THF, a transformation from an unstable concentration oscillation to a stable one occurs. The temperature oscillations show a significant change of the nature of 2**A**(E)
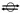
**B**(E) by a small change in the water content from 30 to 33%. A possible explanation of this switching phenomenon can be provided by a structural change of the aqueous solvents between 30 and 33% water content, which affects the interactions between water molecules and then the interactions between TEG groups and water molecules. Reversibility of the switching phenomenon is determined by different experiments [57]. Concentration oscillations induced by temperature oscillations can be used to sense environmental changes by reactions.

## 8. Conclusions

Temperature oscillations can affect behaviors of living things. It is considered that the environmental temperature oscillations are converted into concentration oscillations by chemical reactions. Often, a subtle temperature oscillation can induce significant biological responses, and amplification or positive feedback may be involved in such chemical reactions. Effect of temperature on chemical reactions has generally been studied under fixed temperature (isothermal) conditions. However, effect of temperature oscillation with periodic temperature increase and decrease has not systematically been studied. Such a study on chemical reactions can promote our understanding on the biological events such as flower blossom in spring and circadian rhythms in a day. This article attempted to discuss the effect of temperature oscillations on chemical reactions involving amplification through self-catalytic reactions.

Triangle temperature oscillations induce diverse stable and unstable concentration oscillations when processed by chemical reactions involving self-catalytic reactions. Stable and unstable concentration oscillations are phenomena in which repeated cycles provide the same and different amplitudes, frequencies, periods, and waveforms, respectively. Under equilibrium, a stable concentration oscillation uniquely occurs by a temperature oscillation. In contrast, stable and unstable concentration oscillations occur by reversible nonequilibrium chemical reactions. Delay in concentration oscillations versus temperature oscillations provides different pathways of chemical reactions during cooling and heating, which exhibit delay hysteresis. When the chemical reactions involve amplification through self-catalytic reactions, complex concentration oscillations with amplify hysteresis appear.

Stable and unstable concentration oscillations have been described on the basis of the classifications according to their waveforms in concentration/time profiles, the shapes of hysteresis curves in concentration/temperature profiles, the nature of self-catalytic reactions, and their relationships with the equilibrium curve. Unstable concentration oscillations are transformed into stable concentration oscillations by repeated cycles, and the processes are analyzed using the classifications. Notable nonequilibrium phenomena appear, which include equilibrium intersecting, equilibrium sliding, resonance phenomenon, and transformation from a stable concentration oscillation to an unstable one.

**Figure 28:**
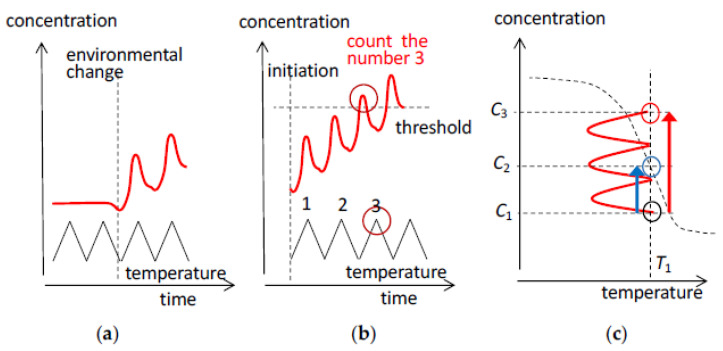
Possible applications of unstable concentration oscillations (**a**) for sensing of an environmental change, (**b**) to count the numbers three, and (**c**) for the enhancement of efficiency of a synthetic chemical reaction, which provides a concentration of the product beyond that of equilibrium. Red and blue arrows in (**c**) indicate chemical yields of a chemical reaction beyond and under equilibrium, respectively; black lines in (**a**,**b**) indicate temperature oscillations.

Possible applications of stable and unstable concentration oscillations are briefly noted below, which exhibit high sensitivity to temperature oscillations owing to self-catalytic reactions. (1) Stable concentration oscillations SAO-1 and SAO-2 may be used for drug delivery. Consider a system such as liposomes containing the oligomers, which can release a drug upon increase in double-helix content. A significant increase in the double-helix upon cooling releases the drug in the night, and the increase upon heating in the morning. (2) A small increase in the environmental temperature by 2 °C may be sensed and initiate unstable concentration oscillation UAN-1, as described in Section 5.2 (Figure 28a). Once initiated, the concentration of double-helix significantly increases regardless of cooling and heating, which may be used to induce a strong response against a small temperature change. (3) Unstable concentration oscillations UAN-2 and UAN-3 may be used for the function to count the numbers (Figure 28b) [44]. The number three, such as 3 days, can be counted when a concentration exceeds a threshold after three cycles of an unstable concentration oscillation.

Concentration oscillations can be employed also to enhance efficiency of a synthetic chemical reaction when the chemical reaction coupled with amplification can proceed beyond equilibrium. Consider a chemical reaction starting from a concentration *C*_1_ of the product at temperature *T*_1_ (Figure 28c, black circle)_._ An unstable concentration oscillation UAN-1 with equilibrium sliding induces the chemical reaction to proceed beyond the equilibrium providing a concentration *C*_3_ (Figure 28c, green circle). Note that *C*_3_ is higher than the concentration *C*_2_, which is obtained by achieving an equilibrium (Figure 28c, blue circle). Then, the chemical yield can exceed that achieved at an equilibrium (Figure 28c, red and blue arrows).

## Figures and Tables

**Table 1:** Classifications of stable and unstable concentration oscillations. S and U indicate stable and unstable concentration oscillations, respectively; E indicates equilibrium; D and A indicate delay and amplify hysteresis, respectively; O, I, and N indicate equilibrium overlapping, equilibrium intersecting, and equilibrium noncontact, respectively.

Stable/Unstable Concentration Oscillations	Waveform	Hysteresis	Symbol
Stable concentration oscillations			
under equilibrium			
	sinusoidal	–––––	SE-1
	triangle	–––––	SE-2
with delay hysteresis			
	normal	normal	SD-1
	sinusoidal	oval	SD-2
with amplify hysteresis involving equilibrium overlapping			
	sinusoidal	semi-normal	SAO-1
	retarded	inflation	SAO-2
	retarded	inflation	SAO-3
		(equilibrium crossing)	
	frequency-doubled	figure-eight	SAO-4
with amplify hysteresis involving equilibrium intersecting			
	sinusoidal	semi-normal	SAI-1
	retarded	inflation	SAI-2
	frequency-doubled	figure-eight	SAI-3
	sinusoidal	long oval	SAI-4
		(equilibrium touching)	
with amplify hysteresis involving equilibrium noncontact			
	retarded	inflation	SAN-1
	sinusoidal	oval	SAN-2
Unstable concentration oscillations			
with amplify hysteresis involving equilibrium noncontact			
	wrinkled	zigzag	UAN-1
	gradient sinusoidal	loop	UAN-2
	gradient retarded	swing	UAN-3
with amplify hysteresis involving equilibrium intersecting (equilibrium sliding)			
	wrinkled	zigzag	UAI-1
	gradient frequency-doubled	twisted loop	UAI-2
